# *Streptococcus pneumoniae* in the heart subvert the host response through biofilm-mediated resident macrophage killing

**DOI:** 10.1371/journal.ppat.1006582

**Published:** 2017-08-25

**Authors:** Anukul T. Shenoy, Terry Brissac, Ryan P. Gilley, Nikhil Kumar, Yong Wang, Norberto Gonzalez-Juarbe, Whitney S. Hinkle, Sean C. Daugherty, Amol C. Shetty, Sandra Ott, Luke J. Tallon, Jessy Deshane, Hervé Tettelin, Carlos J. Orihuela

**Affiliations:** 1 Department of Microbiology, The University of Alabama at Birmingham, Birmingham, AL, United States of America; 2 Department of Microbiology, Immunology, and Molecular Genetics, The University of Texas Health San Antonio, San Antonio, TX, United States of America; 3 Department of Microbiology and Immunology, Institute for Genome Sciences, University of Maryland School of Medicine, Baltimore, MD, United States of America; 4 Division of Pulmonary, Allergy & Critical Care Medicine, The University of Alabama at Birmingham, Birmingham, AL, United States of America; University of Birmingham, UNITED KINGDOM

## Abstract

For over 130 years, invasive pneumococcal disease has been associated with the presence of extracellular planktonic pneumococci, i.e. diplococci or short chains in affected tissues. Herein, we show that *Streptococcus pneumoniae* that invade the myocardium instead replicate within cellular vesicles and transition into non-purulent biofilms. Pneumococci within mature cardiac microlesions exhibited salient biofilm features including intrinsic resistance to antibiotic killing and the presence of an extracellular matrix. Dual RNA-seq and subsequent principal component analyses of heart- and blood-isolated pneumococci confirmed the biofilm phenotype *in vivo* and revealed stark anatomical site-specific differences in virulence gene expression; the latter having major implications on future vaccine antigen selection. Our RNA-seq approach also identified three genomic islands as exclusively expressed *in vivo*. Deletion of one such island, Region of Diversity 12, resulted in a biofilm-deficient and highly inflammogenic phenotype within the heart; indicating a possible link between the biofilm phenotype and a dampened host-response. We subsequently determined that biofilm pneumococci released greater amounts of the toxin pneumolysin than did planktonic or RD12 deficient pneumococci. This allowed heart-invaded wildtype pneumococci to kill resident cardiac macrophages and subsequently subvert cytokine/chemokine production and neutrophil infiltration into the myocardium. This is the first report for pneumococcal biofilm formation in an invasive disease setting. We show that biofilm pneumococci actively suppress the host response through pneumolysin-mediated immune cell killing. As such, our findings contradict the emerging notion that biofilm pneumococci are passively immunoquiescent.

## Author summary

Since its discovery in 1881, invasive disease caused by *Streptococcus pneumoniae*, the leading cause of community-acquired pneumonia and a prototypical extracellular pathogen, has been tied exclusively to the planktonic phenotype, i.e. individual diplococci or short chains. Herein, we report that heart-invaded pneumococci can instead replicate intracellularly and transition into an immunoquiescent biofilm. Using dual RNA-seq technology we capture the complete gene expression profile of *S*. *pneumoniae* within infected hearts as well as the bloodstream. In doing so, we affirm that pneumococci within the heart are indeed forming biofilms and identify virulence determinants that play important roles *in vivo*. Accordingly, we identify a novel role for the genomic island Region of Diversity 12 in promotion of biofilm formation, virulence, and dampening of the host response. We subsequently show that biofilm pneumococci prevent immune cell infiltration into the heart not by passive means but instead through enhanced fratricide-mediated release of the toxin pneumolysin that kills resident macrophages, pre-empting their response. Collectively our manuscript describes a novel site, i.e. intracellular; previously unreported growth phenotype during invasive disease, i.e. biofilm formation; and counter-intuitive molecular mechanism, i.e. resident macrophage killing; for how pneumococci establish themselves in the heart without inflammation.

## Introduction

Hospitalization for community-acquired pneumonia (CAP) is an established risk factor for adverse cardiac events that includes heart failure, arrhythmia, and infarction; with as many as one-in-four adults hospitalized for CAP experiencing some form of pneumonia-associated adverse cardiac event (PACE)[1, 2]. *Streptococcus pneumoniae* (the pneumococcus), the leading cause of CAP [3], has been directly linked to PACE. In 2007, Musher *et al*. reported that one in five individuals hospitalized for pneumococcal pneumonia experienced PACE and these individuals had four-fold greater mortality than those with pneumococcal pneumonia alone [4]. More recently, Eurich *et al*. reported that pneumococcal pneumonia was specifically associated with greater incidence of heart failure, even during convalescence, and this persisted for a period of up to 10 years [5]. Thus, clinical studies strongly imply that some form of cardiac damage is incurred during invasive pneumococcal disease (IPD).

During IPD, circulating *S*. *pneumoniae* are capable of binding to the vascular endothelium and translocating into the heart [6]. Within the myocardium, invaded pneumococci form what we have termed “cardiac microlesions”, i.e. non-purulent pockets of pneumococci that are typically adjacent to blood vessels. Cardiac microlesions have been shown to disrupt normal electrophysiology and impair contractile function [6–8]. In antibiotic rescued animals, including non-human primates with experimental pneumococcal pneumonia, tissue damage associated with cardiac microlesions resulted in *de novo* scar formation [6, 9]. Thus, the acute and long-lasting damage caused by heart-invaded *S*. *pneumoniae* is one explanation for PACE and the adverse cardiac events that occur thereafter. Pertinent to this study, the cholesterol-dependent pore-forming toxin produced by *S*. *pneumoniae*, pneumolysin (Ply), has been shown to play an important role in cardiomyocyte and infiltrated macrophage killing [6–8]. Nonetheless and despite all these advances in knowledge, mechanisms by which pneumococci establish themselves within the myocardium without innate immune cell recognition remains unknown.

In stark contrast to what occurs during pneumonia and IPD, *S*. *pneumoniae* forms biofilms in the nasopharynx during colonization and within the middle ear during otitis media [10, 11]. Biofilms are surface-attached communities of bacteria encased within an extracellular matrix (ECM)[12–15]. In the nasopharynx, biofilm growth of *S*. *pneumoniae* confers resistance to desiccation and the host immune response [10]. The slower metabolic rate of bacteria in biofilms also confers intrinsic resistance to antibiotic killing, which helps to explain the recalcitrance of otitis media to treatment [11]. Importantly, a growing body of literature demonstrates that biofilm pneumococci elicit a considerably weaker immune response from host cells when compared to their planktonic or biofilm-dispersed counterparts [15, 16]. It has been speculated that this promotes long-term colonization by delaying the onset of adaptive immunity [10, 17]. However, the mechanisms by which biofilms suppress immune cell recognition have not been described. Finally, a role for pneumococcal biofilms during IPD has not been reported. This has instead been attributed to the planktonic phenotype which due to reduced surface area better evade stochastic C3b complement deposition and resultant opsonophagocytic killing [18].

Herein we report that heart-invaded pneumococci present within the myocardium replicate within cellular vesicles and over time transition to a mature biofilm. This is the first report of intracellular replication of *S*. *pneumoniae* or pneumococcal biofilm formation in an invasive disease context. Using dual RNA-seq analysis we show that pneumococci within the heart are highly distinct from their circulating counterparts and that virulence gene expression is highly anatomical-site specific; this has key implications on vaccine design. We identify a previously unappreciated genomic island that promotes biofilm formation *in vivo* and show that heart-invaded biofilm pneumococci establish their non-inflammogenic profile not through passive immunoquiescence as previously speculated, but instead via the rapid killing of cardiac macrophages due to enhanced pneumolysin release. These studies advance our understanding of pneumococcal pathogenesis, shed light on the basis of cardiac damage, and reveal a new role for biofilms and pneumolysin during pneumococcal infection.

## Results

### Pneumococci within cardiac microlesions exist as biofilms

**Fig 1:**
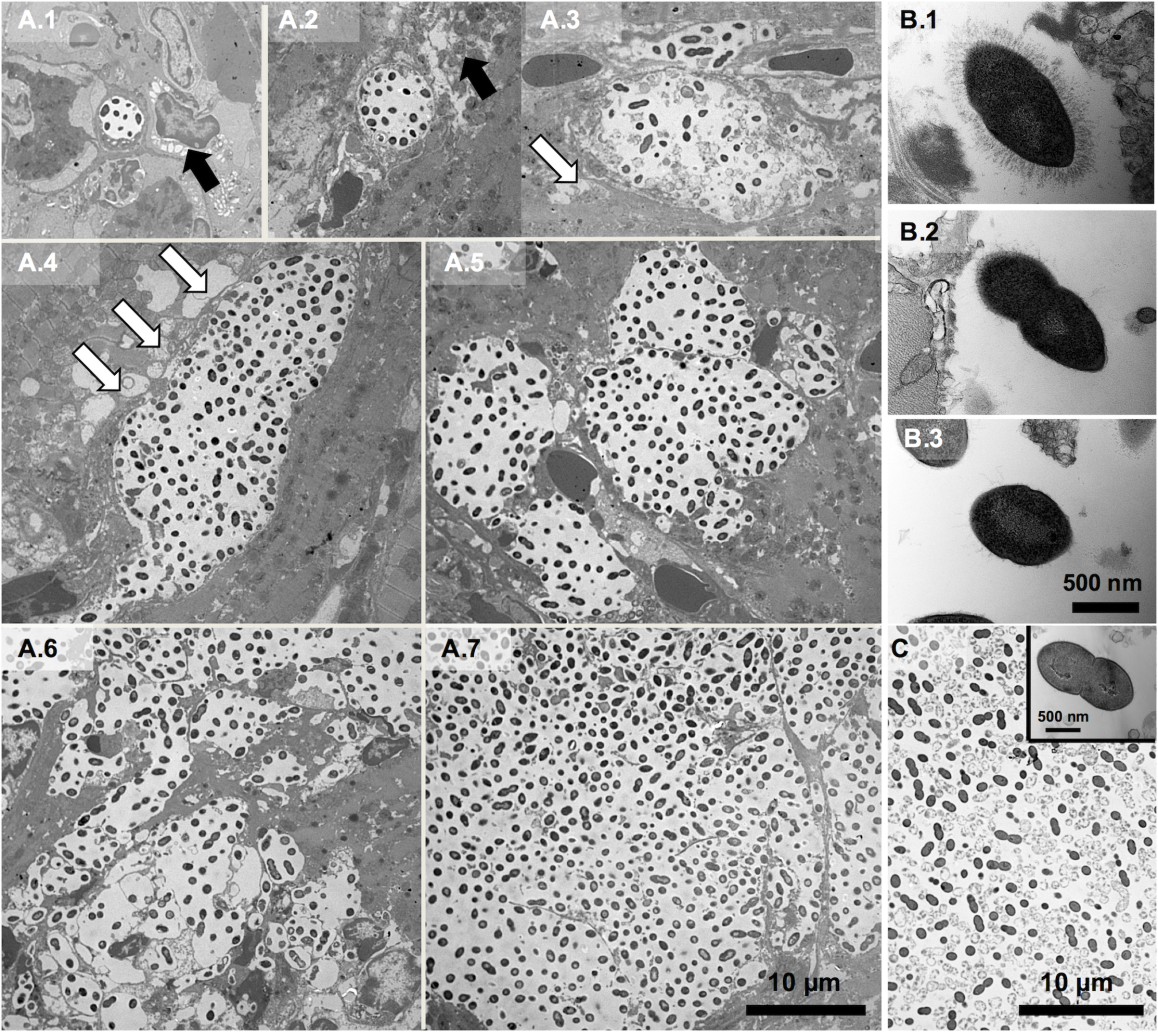
Morphogenesis of cardiac microlesions. **(A)** Representative transmission electron microscopy (TEM) images of cardiac sections (magnification: 2,500X) from BALB/cJ mice infected with *S*. *pneumoniae* strain TIGR4 between 24 and 42 hours post-infection (n = 12). Panels A.1-7 depict the morphogenesis of cardiac microlesions beginning as pneumococci-containing microscopic vesicles within the myocardium. Hydropic degeneration (black bold arrows) and mitochondrial damage as evidenced by swelling (white arrows) adjacent to cardiac microlesions are evident. Images that are the most representative of what occurs during individual microlesion development are shown. The images do not necessarily depict the overall course of infection in a mouse which is mixed with different sized microlesions at late timepoints. **(B)** Representative high power (60,000X) TEM images of pneumococci within microlesions show heterogeneous capsule expression: (B.1) pneumococci within smallest vesicles surrounded by myocardium; (B.2) pneumococci at the periphery of larger microlesions; (B.3) pneumococci within the center of a larger microlesion. **(C)** Representative TEM image of TIGR4 within a 48-hour old static biofilm (n = 3) grown in a 6-well plate (3,000X). **Inset,** Representative high power (60,000X) TEM image of biofilm-pneumococci.

To determine the morphogenesis of microlesions, heart sections from mice infected with *S*. *pneumoniae* serotype 4, strain TIGR4 were examined by transmission electron microscopy (TEM) **(Fig 1A**). The smallest assemblages of pneumococci that could be detected between 18 and 42 hours post infection (hpi) were consistently within clear, spherical, and discrete intracellular vesicles 4–8 μm in diameter each containing 5–10 electron dense diplococci spaced 1–2 μm apart. Pneumococci-filled vesicles were frequently adjacent to swollen mitochondria and alongside or within areas of the cardiomyocytes undergoing hydropic degeneration. All vesicles, even those containing hundreds of pneumococci, had equidistantly spaced diplococci with well-defined separation from the host cytoplasm. The largest microlesions appeared to be the result of bacterial replication along with the expansion, budding, and merging of smaller vesicles. Larger microlesions also had remnants of vesicular membrane present throughout and were consistently associated with cellular debris both within the vesicles and on their periphery. In all instances, larger microlesions were not associated with immune cells.

Considerable bacterial heterogeneity was evident within the larger microlesions. We observed an accumulation of dead, i.e. ghost pneumococci (**S1 Fig**), and differences in regards to the presence of capsular polysaccharide. While pneumococci within the smallest vesicles exhibited uniform capsule distribution **(Fig 1B.1)**, those at the periphery of the larger microlesions had capsule only at one pole **(Fig 1B.2)**, and those at the center of largest microlesions had little to no detectable capsule present **(Fig 1B.3)**. Strikingly, captured TEM images of TIGR4 cardiac microlesions strongly resembled those previously reported for *in vitro* formed biofilms (**Fig 1C**)[19]. Similarities included the equidistant spacing of pneumococci, the accumulation of ghost cells, and reduced presence of detectable capsule. Of note, serotype 6A, strain 6A-10 did not form cardiac microlesions following mouse challenge, despite the presence of detectable bacteria in the myocardium (**S2A Fig**). TEM imaging of 6A-10 infected hearts instead showed pneumococci within macrophages adjacent to the vasculature (**S2B Fig**).

**Fig 2:**
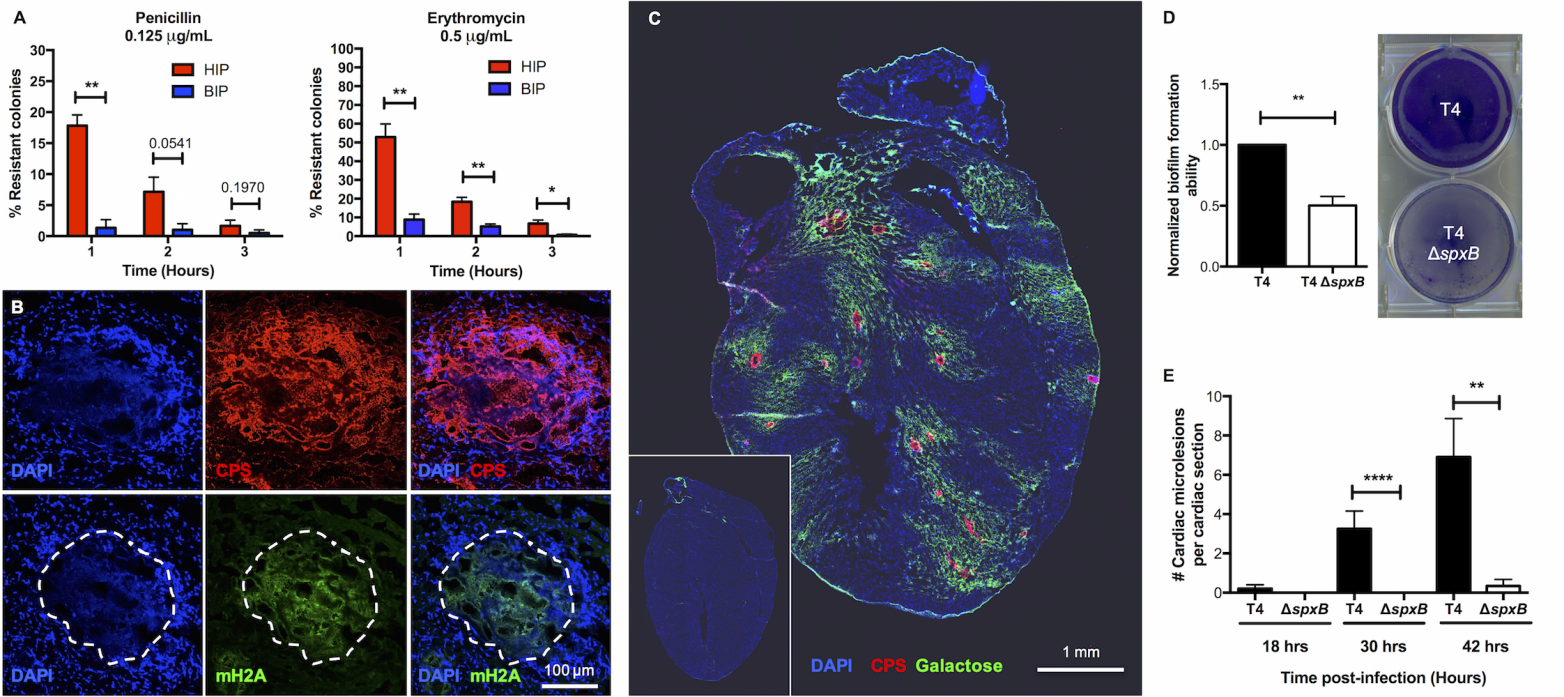
*S*. *pneumoniae* within cardiac microlesions exhibit biofilm properties. **(A)** *In vitro* antimicrobial tolerance assays for survival of paired blood-isolated pneumococci (BIP) and heart-isolated pneumococci (HIP) from the same mouse (n = 6). Tolerance to penicillin and erythromycin killing at the designated concentration was tested. Statistical analysis was performed using Mann-Whitney test. **(B)** Representative high magnification immunofluorescent microscopy images of cardiac microlesions showing presence of biofilm extracellular matrix components: nucleic acids (stained with DAPI [DAPI], *blue*), capsule (stained with anti-serotype 4 capsule antibody [CPS], *red*), and mouse extracellular DNA (stained with anti-mH2A histone antibody [mH2A], *green*). A minimum of 5 stained heart sections were examined. **(C)** Representative tile-stitched image of whole heart sections from TIGR4 infected mice (n = 5). The cardiac sections (stained with DAPI, *blue*) were probed for TIGR4 (*red*), using serotype 4 capsule polysaccharide antisera, and for exposed galactose residues (*green*), using fluorescein labeled *Erythrina crystagalli* lectin, within the heart. **Inset,** Representative tile-stitched image of an uninfected control heart (n = 3). **(D)** Static biofilm-forming ability of TIGR4 (T4) and T4 *ΔspxB* was assessed in 48-hour 6-well plate model (n = 3 biological replicates, each with 2 technical replicates). Biofilm biomass was measured using crystal violet staining. Statistical analysis was performed using Student’s *t*-test. **(E)** Average number of cardiac microlesions detected per cardiac section in T4 and T4 Δ*spxB* infected mice post infection. For each time point, cardiac sections from at least 5 mice were examined. Averages were calculated by enumerating the number of microlesions in 3 non-adjacent cardiac sections. Statistical analysis was performed using Mann-Whitney test. *P* value: * ≤ 0.05, ** ≤ 0.01, **** ≤ 0.0001; data are represented as mean ± SEM.

Given the morphological similarities of TIGR4 within mature cardiac microlesions to those within *in vitro* biofilms, we tested the former for salient biofilm properties. Heart-isolated pneumococci (HIP) were intrinsically resistant to antimicrobial killing; a phenotype absent in blood-isolated pneumococci (BIP) from the same mouse (**Fig 2A**). This resistance to antimicrobials was lost following >1 hour of HIP outgrowth in THY. We also detected extracellular DNA, an established biofilm ECM component [13], within cardiac microlesions (**Fig 2B, S3 Fig**). In the nasopharynx, the absence of glucose and presence of neuraminidase-exposed terminal galactose on mucosal epithelial cells has been shown to promote pneumococcal biofilm formation by de-repressing Streptococcal pyruvate oxidase (SpxB)-mediated metabolism. SpxB has also been demonstrated to be required for *S*. *pneumoniae* biofilm formation within the nasopharynx [20]. Staining of control and infected heart sections with a fluorescent lectin specific for terminal galactose showed this carbohydrate was exposed only in areas of the heart immediately adjacent to cardiac microlesions (**Fig 2C**). What is more, a TIGR4 *spxB* deficient mutant failed to form biofilms *in vitro* (**Fig 2D**) and cardiac microlesions (**Fig 2E**) *in vivo* despite causing sustained bacteremia for 42 hours and adhering to rat brain capillary endothelial cells, i.e. RBCEC6 cells, *in vitro* at normal levels **(S4A and S4B Fig)**. Based upon this collective body of evidence we conclude that cell invaded TIGR4 replicate within small vesicles and transition to a biofilm during mature cardiac microlesion development. Of note, terminal galactose exposure was not evident in mouse hearts having sterile injury due to myocardial infarct (**S5 Fig**). Thus, the exposure of galactose in areas surrounding microlesions was not due to tissue injury.

### Pneumococci in the heart possess a distinct gene expression profile

**Fig 3:**
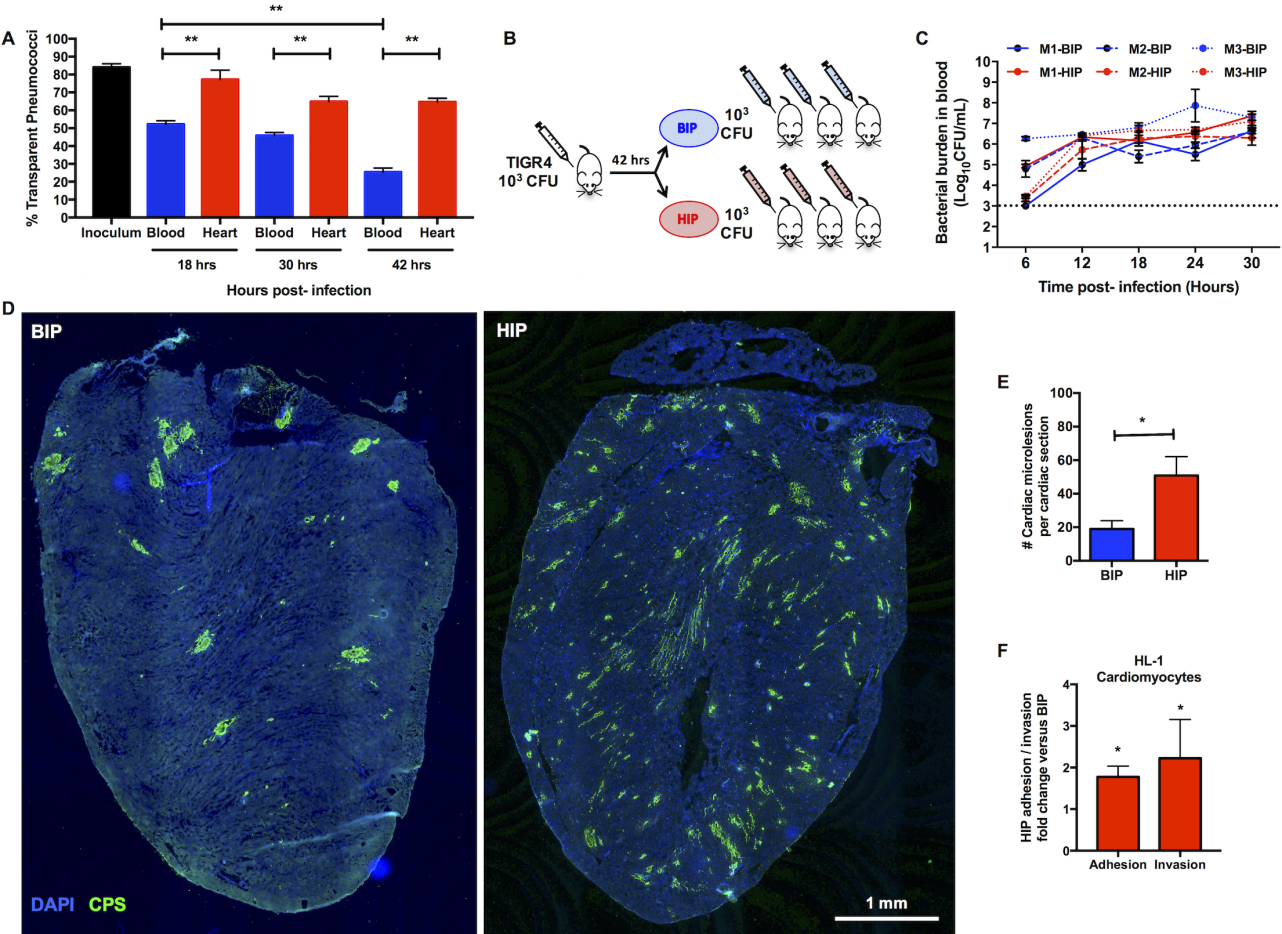
Heart isolated pneumococci exhibit tissue tropism. **(A)** Percentage of pneumococci in the transparent phenotype recovered from the blood (*blue*) and heart (*red*) of infected mice 18, 30, and 42 hours post-infection (n = 5 mice per time point). The TIGR4 parent wildtype strain used as infection inoculum was composed of 84% transparent pneumococci. Multiple group analysis was performed using non-parametric One-way ANOVA (Kruskal-Wallis Test) with Dunn’s multiple comparison test. Comparison of percent transparent colonies of pneumococci isolated from blood or heart at each time points was performed using Mann-Whitney test. **(B)** Schematic representation of the serial infection model used to assess the invasive abilities of paired BIP (*blue*) and HIP (*red*) samples. Three mice were infected with TIGR4 to give 3-paired HIP and BIP samples. Each of these HIP and BIP samples were then used to infect 3 more mice per sample to have a total of 9 HIP-infected mice and 9 BIP-infected mice (3 samples x 3 mice each), i.e. n = 18 total mice. The schematic denotes only one replicate of a 3-part experiment. **(C)** Pneumococcal titers in the blood of mice infected with TIGR4 BIP or HIP over time. The figure denotes blood titers of mice such that each line denotes the mean bacterial titers in blood of the 3 mice that received the same HIP or BIP sample (total n = 9 HIP and BIP infected mice). Paired samples are denoted by line pattern. Statistical analysis was done between groups at each time point using Mann-Whitney test. No statistical significance was observed between the BIP and HIP blood titers at any time point. **(D)** Representative tile-stitched images of whole frozen heart sections from mice infected with BIP and HIP. Cardiac sections (stained with DAPI, *blue*) were probed for TIGR4 using serotype 4 capsule polysaccharide antisera (CPS, *green*). **(E)** Average number of cardiac microlesions per mouse heart section following challenge with BIP (n = 7) or HIP (n = 9). Averages were calculated by enumerating the number of microlesions in 3 non-adjacent cardiac sections. Statistical analysis was performed using Mann-Whitney test. **(F)** Adhesion and invasion of HIP (n = 4) compared to BIP (n = 4) to HL-1 mouse atrial cardiomyocytes *in vitro*. Values are expressed as fold-increase in HIP relative to BIP. Experiments were done using 4 sets of paired HIP and BIP samples collected from 4 individual mice (i.e. 4 biological replicates). Each sample pair was tested against each other using 3 technical replicates on each cell line. The average of each set of technical replicates, was used to create the figure panel and for statistical analysis. Statistical analysis was performed using Mann-Whitney test. *P* value: * ≤ 0.05, ** ≤ 0.01; data are represented as mean ± SEM.

Pneumococci are phase-variable and stochastically alternate between opaque and transparent colony phenotypes. During nasopharyngeal colonization and *in vitro* biofilm formation, the transparent phenotype is selected for due to an enhanced capacity to adhere to surfaces. In the lungs during pneumonia and the bloodstream during IPD, the opaque variant instead dominates due to its enhanced resistance to opsonophagocytic killing [19, 21–23]. Along such lines, the percentage of transparent pneumococci present in blood of TIGR4-infected mice decreased from the inoculum of 84% to 25% during the course of infection **(Fig 3A).** In contrast and within the same animals, the majority of pneumococci isolated from hearts were transparent over time **(Fig 3A)**. We subsequently hypothesized that translocation into the heart was a selective event and required pneumococci with a tissue-tropic phenotype. If true, then mice infected with HIP should experience greater cardiac invasion than those infected with BIP (modeled in **Fig 3B**). Strikingly, HIP-infected animals had a 2.5-fold greater number of cardiac microlesions than their BIP-infected counterparts despite equivalent levels of bacteremia (**Fig 3C–3E**). *In vitro* HIP and BIP exhibited comparable adhesion to and invasion of RBCEC6 cells, yet HIP had enhanced adhesion to and invasion of HL-1 cardiomyocytes **(Fig 3F, S6 Fig).** Thus, TIGR4 isolated from the myocardium were not primed for translocation across vascular endothelial cells and into the heart, but were instead better suited for cardiomyocyte-related interactions.

To elucidate how TIGR4 in the heart differed from those in the bloodstream we performed deep-sequencing of cDNA derived from the infected organs. Total RNA from intact hearts of HIP-infected mice (~10^7−8^ CFU per heart) and pooled blood (~10^7^ CFU/mL) from TIGR4-infected neutropenic mice was used to generate cDNA and ≥300 million RNA-seq reads were performed per biological sample. The normalized number of RNA-seq reads mapping to each TIGR4 gene (RNA reads per kilobase of transcripts per million mapped reads, RPKM) is a direct correlate of individual gene expression levels (**S1 Table**). For pairwise differential expression analyses, RNA-seq reads were first sub-sampled to match the condition with the lowest number of reads; these normalized RPKM values are provided in **S2 Table.** It is of note that this approach yielded RNA reads that corresponded to ~90% of the TIGR4 genome. This approach also yielded an exhaustive in-depth reading of transcripts corresponding to infected mice. All RNA-seq data generated as part of this study is available through the NCBI Gene Expression Omnibus (GEO) database (see methods).

**Fig 4:**
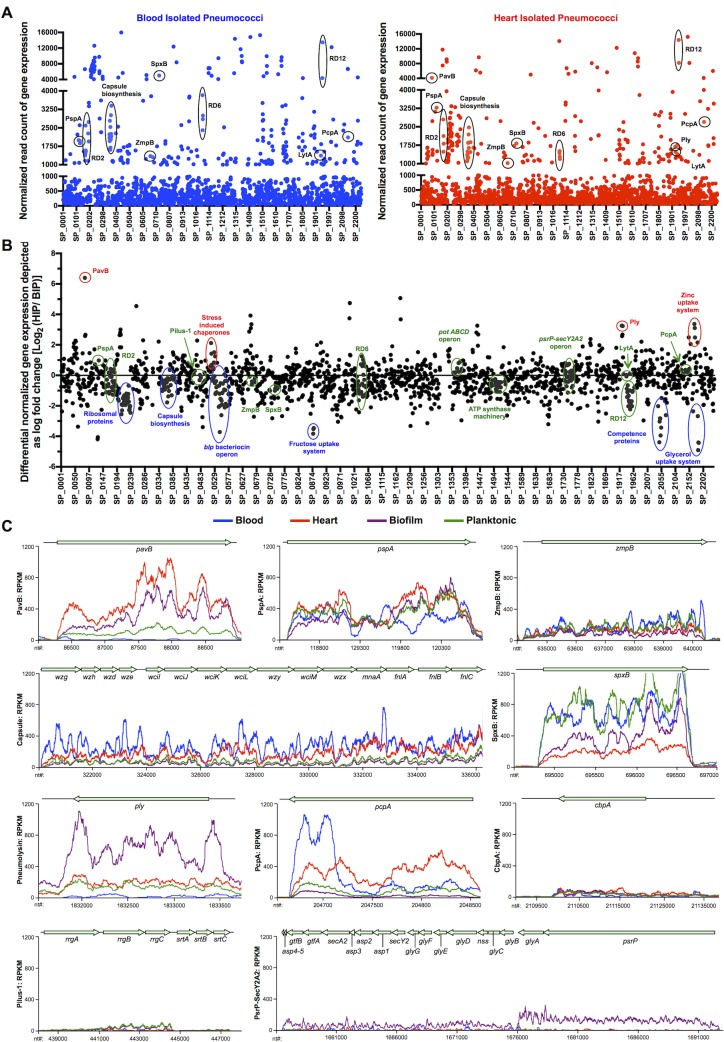
Comparative gene expression analysis of HIP, BIP and pneumococci from *in vitro* biofilm and planktonic pneumococci. **(A)** Dot plot representation of the whole genome transcriptomic profile for blood- isolated pneumococci, BIP (*blue*) and heart-isolated pneumococci, HIP (*red*) showing the average normalized number of RNA-seq reads identified, i.e. gene expression levels mapping to each TIGR4 gene within the blood and the heart. Y-axis denotes normalized expression levels (RPKMs) whereas X-axis denotes location of the genes on the TIGR4 chromosome. Established virulence determinants with expression levels >1000 RNA-seq reads (i.e. corresponding to top 10% of genes with highest expression levels) for BIP and HIP are indicated. **(B)** Dot plot representation of the differential gene expression profile for BIP and HIP spanning the TIGR4 genome. The fold changes are depicted as Log_2_(HIP/BIP). Y-axis denotes log fold changes in gene expression levels whereas X-axis denotes location of the genes on the TIGR4 chromosome. Important differentially up-regulated pneumococcal genes for BIP and HIP are indicated in blue and red respectively. Genes clustered near the X-axis are consistently expressed. **(C)** Curve plot representation of gene expression levels for genes encoding designated pneumococcal virulence determinants in the BIP, HIP, *in vitro* biofilm-, and *in vitro* planktonic- TIGR4 samples. Y-axis denotes normalized expression levels (i.e. RPKMs) whereas X-axis denotes individual nucleotide location (nt coordinates) on the TIGR4 chromosome. Two pooled BIP samples (5 mice per sample), three HIP samples, three *in vitro* biofilms and three *in vitro* planktonic pneumococci samples were tested.

While the results provided in **S1**and **S2 Tables** allow for detailed genome-wide comparisons between the HIP and BIP transcriptomes, herein we focused only on established virulence determinants that would inform us on the pathogenic process (**Fig 4**). Virulence determinants with the highest levels of gene expression (i.e. >1000 RPKMs: corresponding to ~10% of encoded genes) in the heart were: Pneumococcal adhesion and virulence protein B (PavB; SP_0082), Pneumococcal surface protein A (PspA, SP_0117), the capsular polysaccharide biosynthesis locus (SP_0346–0360), Zinc metalloprotease B (ZmpB, SP_0664), SpxB (SP_0730), Ply (SP_1923), Autolysin (LytA, SP_1937), and Pneumococcal choline binding protein A (PcpA, SP_2136) (**Fig 4A**). The high levels of gene expression for these virulence determinants suggest that they play an important role within the heart. In stark contrast, the genes encoding PavB and pneumolysin were negligibly expressed within blood. Notably, the genes encoding Pneumococcal pilus-1 (RlrA pathogenicity islet, SP_0462–0468), Pneumococcal serine-rich repeat protein (PsrP) and its accessory proteins (*psrP-secY2A2*, SP_1755–1772), and Choline binding protein A (CbpA, SP_2190) had minimal expression in both heart and blood. Thus, unequivocal anatomical site-specific differences in virulence gene expression occurred *in vivo*, with some previously identified key determinants having surprisingly low levels of both blood and heart-related transcription (**Fig 4B and 4C**). qRT-PCR of a 69-gene panel validated these RNA-seq results (**S7 Fig**).

**Fig 5:**
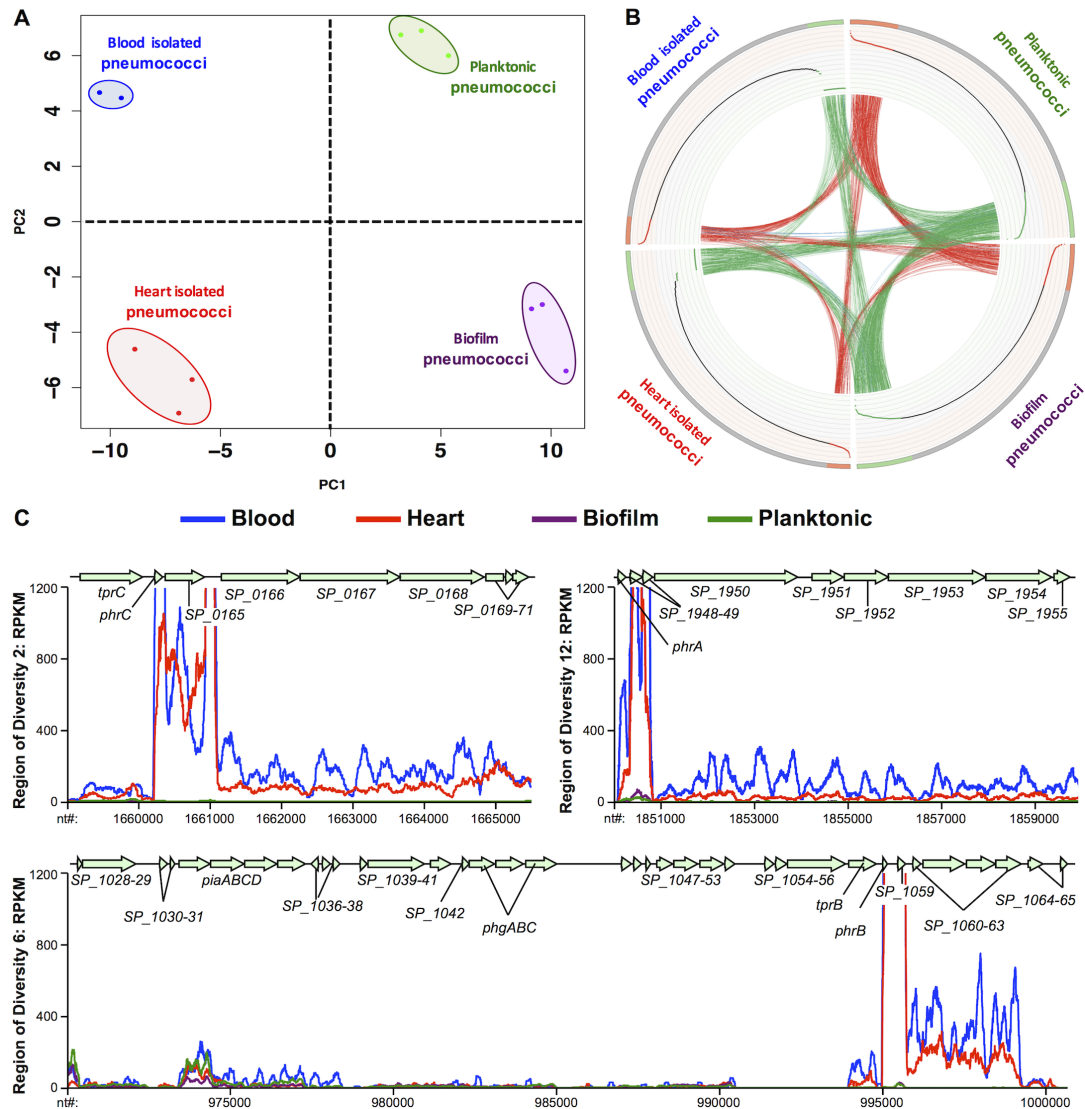
Pneumococcal transcriptome in the heart is distinct from the blood and *in vitro* conditions. **(A)** Two-dimensional (PC1-PC2) principal component analysis on transcriptomes of BIP, HIP, *in vitro* biofilm-, and *in vitro* planktonic TIGR4 samples. PC1 separates the *in vivo* conditions from the *in vitro* conditions. PC2 separates biofilm conditions from planktonic conditions. **B)** Whole transcriptome comparisons for differentially expressed genes under the above conditions presented as circular plots (Circos software, see methods). **Ideogram (outer rim)**: Each quadrant denotes one condition with 1,969 genes ranked by their expression. Red denotes highly expressed genes, gray indicates intermediate expressed genes, and green indicates low expressed genes for each condition. Genes with counts of 0 reads across all samples were excluded. **Scatter Plot (inner rim)**: The scatter plot illustrates the Log_10_ (Count Per Million-mapped- reads) values for each gene in each condition ranging from -2 (inner) to 5 (outer) in steps of 0.5. Red dots are highly expressed genes; black dots are intermediate expressed genes and green dots are low expressed genes. **Links (interior)**: Red arcs link genes with high expression in both conditions. Green arcs connect genes with low expression in both conditions. Blue arcs link genes with low expression in one condition and high expression in another condition. A higher density of connectivity for links of same color indicates transcriptomic similarities within the pneumococcal populations isolated from the tested conditions. (**C)** Curve plot representation of gene expression levels for Regions of Diversity RD2, RD6 and RD12 in the BIP, HIP, *in vitro* biofilm-, and *in vitro* planktonic- TIGR4 samples. Y-axis denotes normalized expression levels (i.e. RPKMs) whereas X-axis denotes individual nucleotide location (nt coordinates) on the TIGR4 chromosome. RD corresponds to Regions of Diversity as determined by Tettelin and Hollingshead [24]. The Tpr/Phr peptide quorum sensing-signaling cassettes within the RDs are indicated as determined by Hoover et al [46]. Two pooled BIP samples (5 mice per sample), three HIP samples, three *in vitro* biofilms and three *in vitro* planktonic pneumococci samples were tested.

We subsequently performed principal component analysis (PCA) using our *in vivo* RNA-seq data and transcriptome data obtained from *in vitro* planktonic- and *in vitro* biofilm-grown pneumococci (**Fig 5A**). Replicates of the same condition (same color) clustered most closely together on the PCA plot as expected. The second principal component, PC2 (Y-axis) separated HIP and *in vitro* biofilm pneumococci (bottom of the plot) from the BIP and *in vitro* planktonic conditions (top of the plot), confirming that the HIP harbored gene expression features more similar to *in vitro* biofilm pneumococci than to *in vitro* planktonic pneumococci. Conversely, pneumococci in the blood were more similar to *in vitro* planktonic pneumococci than *in vitro* biofilm pneumococci. In addition, pneumococci *in vivo* also harbored different sets of gene expression features that clearly distinguished them from their *in vitro* counterparts by virtue of their separation along PC1 (X-axis, left and right sides of the plot, respectively); circos analyses of the RNA-seq data confirmed these relationships **(Fig 5B)**. The gene expression profiles that governed the separation of biofilm-planktonic **(S8 Fig)** and *in vitro*-*in vivo* populations **(S9 Fig)** are provided. Cumulatively, these findings show that *in vivo* gene expression profile is anatomical site-specific and drastically different from *in vitro*.

### RD12 is required for biofilm formation *in vitro* and reduces cardiopathology

Driving the disparity between *in vivo* and *in vitro* transcription profiles were genes encoded within Region of Diversity (RD)2 (SP_0163 to SP_0171), the second half of RD6 (SP_1057 to SP_1065), and RD12 (SP_ 1947 to SP_1955), which were only expressed *in vivo* (**Fig 5C, S9 Fig**). RDs are horizontally acquired genomic islands not present in all pneumococci, many of which have been shown to contribute to virulence [24, 25]. We did not focus subsequent attention on RD6 as prior studies have shown that this 27-kb pathogenicity island encodes *piaABCD*, the pneumococcal iron-acquisition operon, and *phgABC*, the pneumococcal hyperosmotic growth operon; both of which were required for pneumococcal survival in the blood [26–28].

**Fig 6:**
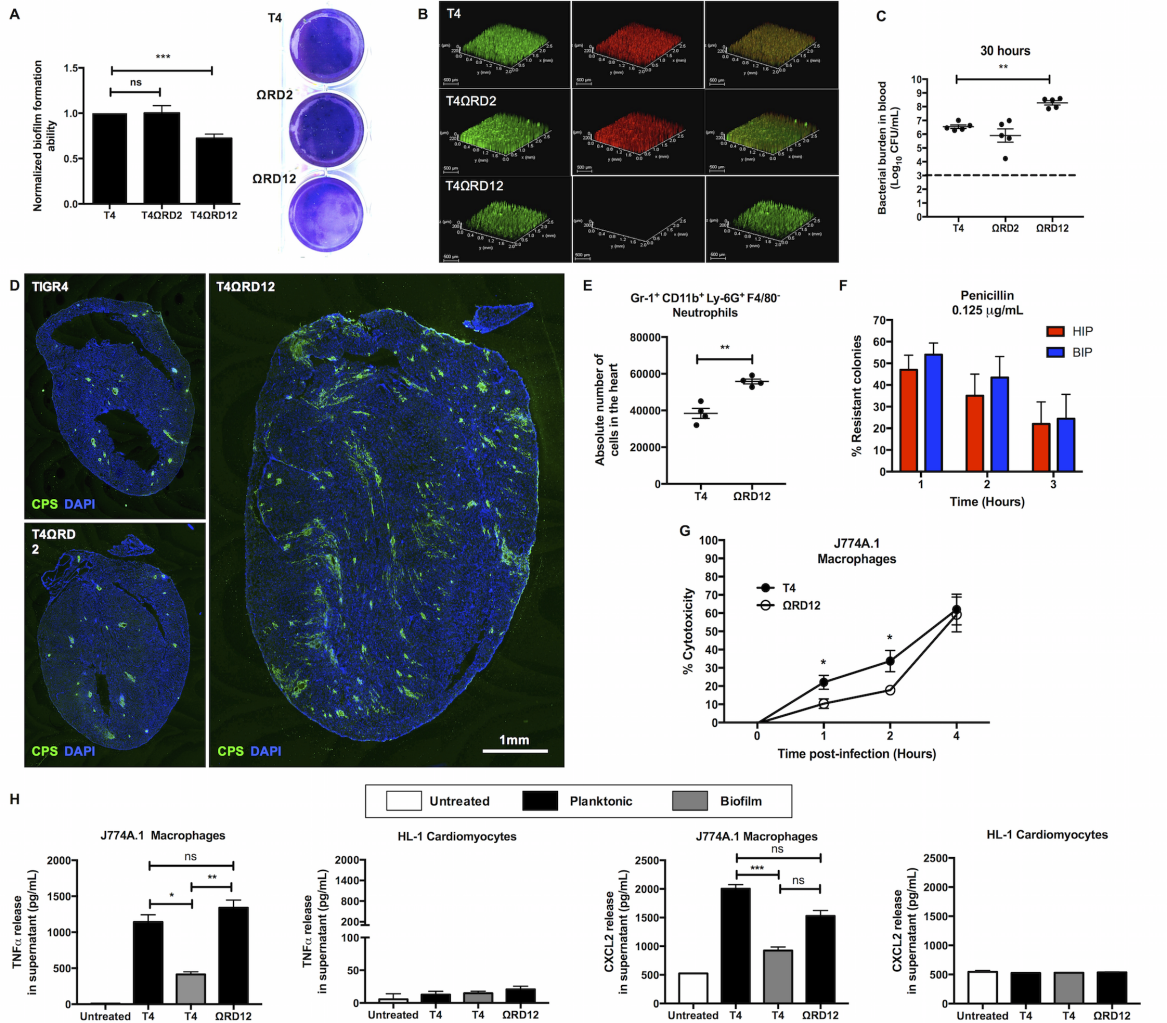
Deletion of RD12 abrogates TIGR4 biofilm formation *in vitro*, results in a pro-inflammogenic phenotype *in vivo*, and elicits a stronger immune response from macrophages. **(A)** Static biofilm-forming ability of isogenic RD2 (T4ΩRD2) and RD12 (T4ΩRD12) deficient mutants relative to TIGR4 (T4) was assessed in a 48-hour 6-well polystyrene plate model (n = 7 experiments). Biofilm biomass was measured using crystal violet staining. Statistical analysis was performed using Student’s *t*-test. Shown is a representative image of crystal violet stained biofilms. **(B)** Representative Z-stacked images of TIGR4, T4ΩRD2, and T4ΩRD12 biofilms grown on coverslips for 24 hours. Biofilms were examined for viability using Live/Dead staining. Whole (live and dead) bacterial biomass stain green whereas non-viable bacteria stain red (n = 3 experiments). **(C)** Pneumococcal titers in the blood of mice (n = 5 per group) infected with TIGR4 (T4), T4ΩRD2 (ΩRD2), or T4ΩRD12 (ΩRD12) 30 hours post-infection. Mann-Whitney test comparing the mutant strain titers to the wildtype TIGR4 titers was performed. **(D)** Representative immunofluorescent stained images of cardiac sections from mice infected with TIGR4 (T4), T4ΩRD2 (ΩRD2), or T4ΩRD12 (ΩRD12) 30 hours post infection (n = 5 mice per group). Cardiac sections were stained using serotype 4 capsule polysaccharide antisera (*green*) and DAPI (*blue*). **(E)** Absolute numbers of infiltrated neutrophils in hearts of mice (n = 5 per group) infected with TIGR4, and T4ΩRD12 (ΩRD12) 30-hours post-infection. Neutrophils were identified as Gr-1^+^CD11b^+^Ly-6G^+^F4/80^-^ cells. Statistical analysis was performed using student’s *t-*test. **(F)** *In vitro* antimicrobial tolerance assays for survival of paired blood-isolated pneumococci (BIP) and heart-isolated pneumococci (HIP) from T4ΩRD12 infected mice (n = 5). Tolerance to penicillin and erythromycin killing at the designated concentration was tested. Statistical analysis was performed using Mann-Whitney test. No statistically significant differences were observed. **(G)** LDH release cytotoxicity assay of J774A.1 macrophages challenged with equal biomass of TIGR4 (T4) and T4ΩRD12 (ΩRD12) as determined at 0, 1, 2, 4 hours post-infection (n = 3 biological replicates, each with 3 technical replicates). Statistical analysis for comparisons of cytotoxicity at each time point was performed using Mann-Whitney test. **(H)** TNFα and CXCL2 production by J774A.1 macrophages and HL-1 cardiomyocytes following 4-hour exposure to an equal biomass of planktonic TIGR4 (T4), biofilm TIGR4 (T4), or planktonic T4ΩRD12 **(**ΩRD12) (n = 3 biological replicates, each with 3 technical replicates). Statistical analysis was performed using non-parametric One-way ANOVA (Kruskal-Wallis Test). *P* value: * ≤ 0.05, ** ≤ 0.01, *** ≤ 0.001; data are represented as mean ± SEM.

We explored whether RD2 or RD12 impacted the ability of TIGR4 to form biofilms and cardiac microlesions. While deletion of RD2 had no discernible effect, deletion of RD12 abrogated biofilm formation in a two-day polystyrene plate model (**Fig 6A**). RD12 encodes a two-component class II pneumococcal lantibiotic bacteriocin called pneumococcin A1/A2 and its accessory proteins (**S10A Fig**)[29]. As such, we speculated that RD12 might promote fratricide which is an important aspect of biofilm ECM formation [30]. Indeed, the RD12 deficient mutant (T4ΩRD12) exhibited a stark absence of propidium iodide stainable DNA, typically accessible in dead pneumococci, following growth on glass coverslips **(Fig 6B)**. This occurred despite the fact that deletion of RD12 did not have any long-term growth defects or changes in autolysis activity as determined by bile solubility assay (**S10B and S10C Fig**). *In vivo*, mice infected with the RD2 deficient mutant had equivalent levels of bacteria in the blood compared to wildtype infected controls. T4ΩRD12 was however hyper-virulent with ~10-fold higher bacterial titers in blood of infected mice at 30 hours (**Fig 6C**). This was not due to changes in capsule levels **(S11 Fig)**. In stark contrast to wildtype TIGR4, mice infected with T4ΩRD12 demonstrated profuse bacterial dissemination in the heart (**Fig 6D**) accompanied by significantly greater neutrophil infiltration (**Fig 6E**). T4ΩRD12 did not form biofilms in the heart as evidenced by the loss of intrinsic antibiotic resistance in HIP when compared to paired blood isolates (**Fig 6F**).

### Biofilm pneumococci rapidly kill tissue macrophages in a pneumolysin-dependent manner to subvert host immune response

Our results suggest that biofilm formation is in some fashion tied to a muted neutrophil response in the heart. In support of this notion, macrophages exposed to wildtype biofilm TIGR4 produced less chemokine-inducing TNFα and CXCL2 than did macrophages challenged with wildtype planktonic TIGR4 or their biofilm-incapable RD12 deficient counterparts (**Fig 6H, S12A Fig**). HL-1 cardiomyocytes did not produce meaningful TNFα, CXCL1, or CXCL2 (**Fig 6H, S12A Fig**) following TIGR4 challenge.

**Fig 7:**
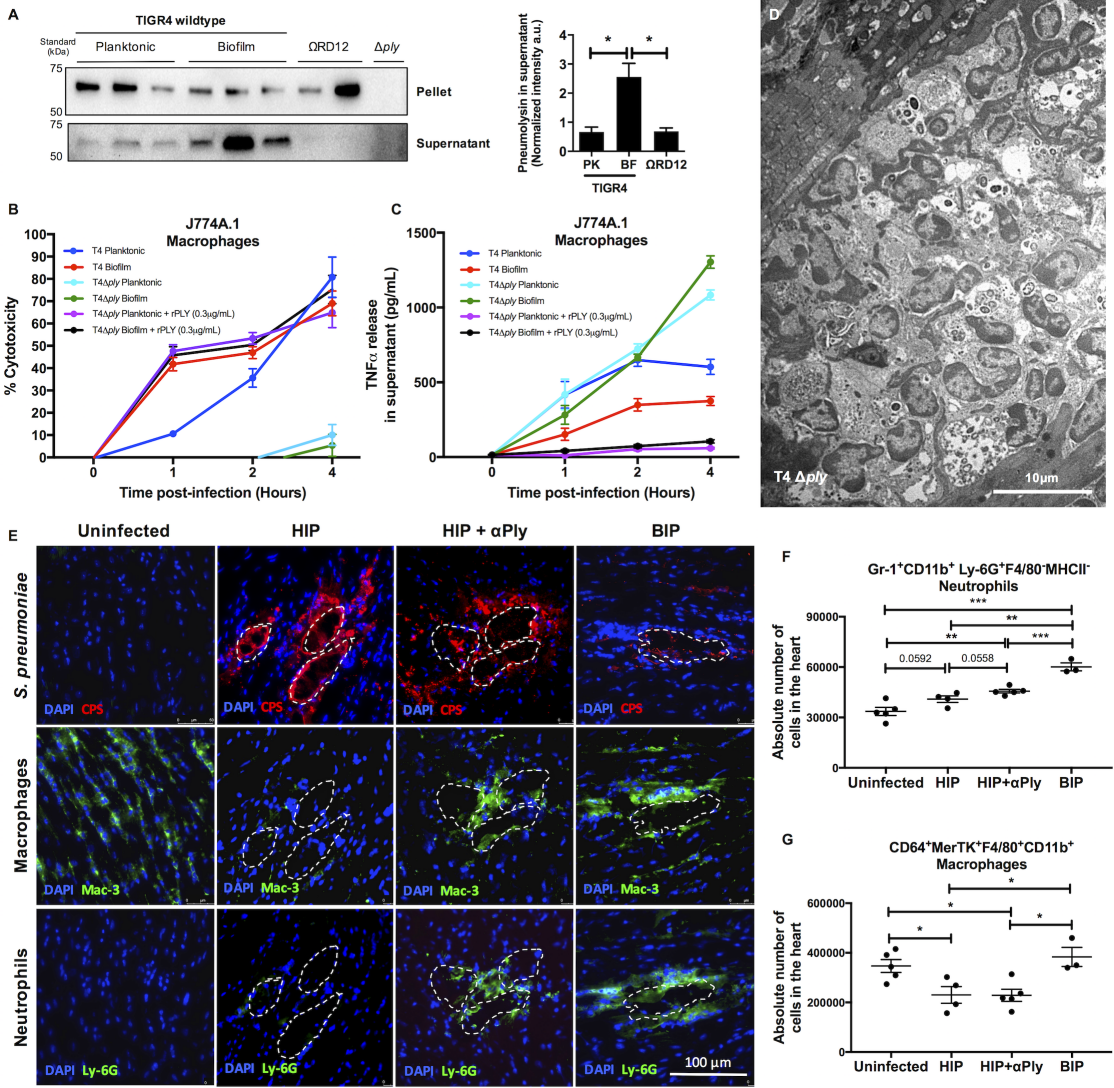
Heart invaded biofilm pneumococci subvert host immune response by releasing pneumolysin and rapidly kill cardiac macrophages. **(A)** Western blots for pneumolysin levels in equal biomass of whole cell lysates (pellets) and supernatants of planktonic- wildtype TIGR4 (n = 3), biofilm- wildtype TIGR4 (n = 3), and planktonic T4ΩRD12 (ΩRD12) (n = 2). An isogenic pneumolysin deficient TIGR4 strain (T4 Δ*ply*) was tested as the negative control. Normalized densitometric quantification of pneumolysin levels in the supernatant is provided. Statistical analysis was performed by comparison supernatant pneumolysin levels from planktonic (PK)- wildtype TIGR4 (n = 3), and planktonic T4ΩRD12 (ΩRD12) (n = 2) to biofilm (BF)- wildtype TIGR4 (n = 3) using Welch’s *t*-test. **(B)** LDH release cytotoxicity assay of J774A.1 macrophages challenged with equal biomass of planktonic-, biofilm- TIGR4 (T4), planktonic-, biofilm- T4 Δ*ply* and planktonic-, biofilm- T4 Δ*ply* complemented with exogenous recombinant pneumolysin (rPLY, 0.3μg/mL) as determined at 0, 1, 2, 4 hours post-infection (n = 3 biological replicates, each with 3 technical replicates). Statistical analysis was performed using ordinary one-way ANOVA. **(C)** TNFα production by J774A.1 macrophages at designated time points following exposure to an equal biomass of planktonic-, biofilm- TIGR4 (T4), planktonic-, biofilm- T4 Δ*ply* and planktonic-, biofilm- T4 Δ*ply* complemented with exogenous recombinant pneumolysin (rPLY, 0.3μg/mL) as determined at 0, 1, 2, 4 hours post-infection (n = 3 biological replicates, each with 3 technical replicates). Statistical analysis was performed using ordinary one-way ANOVA. **(D)** Representative transmission electron microscopy (TEM) image of cardiac sections (magnification: 2,500X) from BALB/cJ mice infected with T4 Δ*ply* 30 hours post-infection (n = 3). **(E)** Representative high magnification immunofluorescent microscopy images of cardiac microlesions from uninfected-, passively immunized (αPly)- and naïve- mice infected with HIP or BIP 30 hours post infection, showing presence of: capsule (stained with anti-serotype 4 capsule antibody [CPS], *red*), cardiac macrophages (stained using anti-Mac-3 antibody [Mac-3], *green*), and infiltrated neutrophils (stained with anti-Ly-6G antibody [Ly-6G], *green*). A minimum of 4 stained heart sections were examined. **(F)** Absolute numbers of infiltrated neutrophils in hearts of uninfected-, passively immunized (αPly)- and naïve- mice infected with HIP or BIP 30 hours post infection. Neutrophils were identified as Gr-1^+^CD11b^+^Ly-6G^+^F4/80^-^ MHC-II^-^ cells. Statistical analysis was performed using student’s *t-*test. **(G)** Absolute numbers of cardiac macrophages in hearts of uninfected-, passively immunized (αPly)- and naïve- mice infected with HIP or BIP 30 hours post infection. Macrophages were identified as CD64^+^MerTK^+^F4/80^+^CD11b^+^ cells. Statistical analysis was performed using student’s *t-*test. *P* value: * ≤ 0.05, ** ≤ 0.01, *** ≤ 0.001; data are represented as mean ± SEM.

Ply is highly inflammatory [31–33]. Therefore, the observation that TIGR4 biofilms were immunoquiescent conflicted with our results that showed *ply* was expressed at higher levels in the heart and within *in vitro* biofilms than blood or *in vitro* planktonic TIGR4, respectively. Subsequent immunoblot analyses confirmed that biofilm TIGR4 released greater amounts of Ply into the supernatant than did planktonic TIGR4 (**Fig 7A**). Explaining this discrepancy, we observed that biofilm TIGR4 killed macrophages faster than planktonic TIGR4 (**Fig 7B**); and that this coincided with a marked reduction in detectable levels of TNFα (**Fig 7C**) and CXCL2 (**S12B Fig**) in supernatants. Planktonic T4ΩRD12 was also less cytotoxic to macrophages than were wildtype pneumococci (**Fig 6G**), consistent with an observed reduction in pneumolysin release (**Fig 7A**), and previously described greater inflammogenic profile in the heart (**Fig 6D and 6E**) and *in vitro* **(Fig 6H)**. Implicating pneumolysin as the principal factor responsible for this phenotype, pneumolysin deficient TIGR4 mutant (T4Δ*ply*) grown as biofilm had negligible cytotoxicity (**Fig 7B, S12C Fig**) and elicited a robust macrophage response (**Fig 7C, S12B Fig**). What is more, complementation of the pneumolysin deficient biofilm-TIGR4 with exogenous pneumolysin restored cytotoxicity to wildtype levels (**Fig 7B**) and severely dampened TNFα and CXCL2 production by challenged macrophages (**Fig 7C, S12B Fig**). Thus *in vitro*, pneumolysin released by biofilm TIGR4 pre-empted macrophage cytokine and chemokine production by their rapid killing. Of note, 6A-10 which naturally lacks RD12, did not show enhanced release of pneumolysin during biofilm growth, and had a comparatively modest enhancement in capability to kill macrophages and subvert cytokine production as a biofilm (**S13 Fig**). This perhaps explains its inability to form cardiac microlesions *in vivo*.

Finally, we examined hearts from TIGR4 and T4Δ*ply* infected mice for differences in cardiac macrophage numbers and neutrophil infiltrates. Unfortunately, drastic differences in bacterial burden between cohorts made direct comparisons between these strains and conditions invalid (**S14A and S14B Fig**). Nonetheless, the rare cardiac microlesion formed within T4Δ*ply* infected mice displayed extensive immune cell infiltration when examined by TEM and immunofluorescent microscopy (**Fig 7D, S14C Fig**). As a work around, we passively immunized mice with neutralizing antibody against Ply and infected mice with wildtype HIP or BIP. Consistent with a prior report [6], antibodies against Ply had no impact on pneumococcal burden in the bloodstream of TIGR4 challenged mice; nor in this instance, within the heart (**S14D and S14E Fig**). Cardiac sections from HIP-infected mice that received Ply antibody stained positively for macrophages and neutrophils in the area immediately surrounding microlesions. In contrast, cardiac sections from mice not receiving antibody lacked the same, indicating immune cell death and/or less neutrophil infiltration (**Fig 7E**). Of note, this robust modulation of immune cells was seen only at the interface of biofilm contact with the host cells in the myocardium. At the gross level and using flow cytometry of whole heart extracts as measure, mice that received Ply antibody had more neutrophils in their hearts than did untreated controls following HIP challenge (**Fig 7F**), whereas the number of cardiac macrophages remained constant (**Fig 7G**). What is more, untreated mice infected with BIP had the greatest number of cardiac macrophages and neutrophils detected by immunofluorescent microscopy and flow cytometry (**Fig 7E–7G, S14F Fig**). Thus, biofilm formation in the myocardium by HIP indeed suppressed subsequent immune cell infiltration and this occurred in a highly focal and Ply-dependent manner.

## Discussion

This is the first report to describe intracellular replication of *S*. *pneumoniae* and to demonstrate an integral role for biofilms during IPD. Together these are a mechanism by which pneumococci establish themselves within the myocardium and subvert their clearance. Our dual RNA-seq approach, in addition to providing insight in regards to the pathogenic process, revealed anatomical-site specific differences in *S*. *pneumoniae* virulence gene expression that may have major implications on antigen selection for future protein-based vaccines. The unexpected observation that biofilm pneumococci in the heart pre-empt immune cell infiltration through rapid resident macrophage killing strongly suggests that the immunoquiescent profile of biofilm attributed to pneumococci at other sites, i.e. nasopharynx, may be through similar means. Thus, this study advances our understanding of pneumococcal pathogenesis and the roles of biofilms and pneumolysin.

TEM examination of infected hearts revealed *S*. *pneumoniae* is capable of intracellular replication within the myocardium. This observation was unexpected since *S*. *pneumoniae* is prototypical for extracellular Gram-positive bacteria [34]. Although observations of intracellular pneumococci within adenoid biopsy specimens from children with otitis media or rhinosinusitis have been reported [35, 36], and pneumococci are known to translocate across vascular endothelial cells within intracellular compartments [37], this is to our knowledge the first report to suggest intracellular replication as an integral step in pneumococcal pathogenesis. Importantly, the exact cell type(s) affected within an infected heart remains to be elucidated. Most probable candidates based on their abundance include cardiomyocytes, resident macrophages, and/or fibroblasts. Moreover, the origin of the early bacteria-filled vesicle is also unclear. The most likely possibilities are: 1) extracellular uptake in a clathrin-coated vesicle from the cell surface [37], or 2) xenophagy, the process by which a cell directs autophagy against an internalized pathogen [38]. Ongoing studies are focused on identifying the specific cells types involved and molecular basis for cardiomyocyte invasion. We propose that intracellular replication allows heart-invaded pneumococci, initially in a planktonic state, to evade innate and adaptive immune mechanisms as they transition into a biofilm.

Bacteria within biofilms are characterized by intrinsic resistance to antibiotic killing, attachment to a surface, and production of an extracellular matrix [12]; all properties observed for pneumococci within infected hearts. *S*. *pneumoniae* biofilms have also been associated with an accumulation of non-viable cells within the biofilm, greater frequency of the transparent phenotype, heterogeneous production of capsule, enhanced adhesiveness to cells, and a requirement for pyruvate oxidase [15, 19, 21, 23]. These features were also observed for pneumococci within infected hearts. Given that pneumococci can be detected within other organs, such as the spleen, following bacteremia [39], it is reasonable to propose that biofilms may be forming in other organs during disseminated infection. Of particular interest are the kidneys, since pneumococcal infection has also been linked to acute kidney injury and long-term dysfunction [40].

Dual RNA-seq is a powerful technique that provided a snapshot of how *S*. *pneumoniae* adapts to and interacts with the host in the heart. For sake of brevity, we do not discuss the host response and limit our discussion to the established virulence determinants that provide insight on the pathogenic process. Ply is a pore-forming toxin that kills cells via necroptosis during microlesion formation [6, 7]. As shown by Shak *et al* and herein, its release by biofilm pneumococci helps the bacterium to form biofilms on the host cell surface [41] and establish residency by killing host tissue-resident macrophages. PavB is a fibronectin-binding MSCRAMM (i.e. microbial surface component recognizing adhesive matrix molecules)[42]. Fibronectin in damaged heart tissue has been conclusively reported and PavB most likely acts as a cardio-adhesin [43]. PspA is the major choline binding protein found on the surface of the pneumococcus and is involved in resistance to complement [44]. Pneumococcal choline binding protein A, PcpA, not to be confused with the adhesin CbpA, has been demonstrated to play a vital role in modulating the host immune response by recruiting myeloid-derived suppressor cells and controlling the inflammatory environment within the lungs during pneumonia [45]. We are currently testing whether this occurs in the heart and is an additional explanation for the lack of immune cells associated with cardiac microlesions. Finally, each of the RDs up-regulated in the heart were associated with a copy of the Tpr/Phr peptide quorum sensing-signaling cassette (SP_0163–0164 in RD2, SP_1057–1058 in RD6, and SP_1946–1947 in RD12)[46]. Other investigators have demonstrated a role for Tpr and its orthologs in biofilm formation and virulence of diverse bacteria [47–49].

The reasons for why an RD12 deficient mutant was hyper-virulent are not fully clear. In the heart, and based upon our *in vitro* results, we propose that RD12 encoded Pneumococcin A contributes towards biofilm formation by inducing fratricide and it is the resultant release of bacterial products including DNA and Ply that helps to establish the ECM [13, 41]. The latter also contributing to the non-inflammogenic biofilm phenotype that was observed. We and others have previously reported that pneumococci within biofilms elicit a muted host response from nasopharyngeal epithelial cells *in vitro* and the airways of challenged mice [15, 16]. As such, we initially hypothesized that the absence of immune cell infiltrates within cardiac microlesions was due to some form of passive immune evasion. Our observation that macrophages responded robustly to pneumococci lacking pneumolysin in biofilms disproved the former. Instead, it became evident that the observed muted cytokine response was due to rapid killing of cardiac macrophages by pneumolysin, thereby pre-empting the host response. Importantly, our previous study i.e. Gilley *et al*. concluded that pneumolysin produced by pneumococci in the heart killed infiltrating monocytes [7]. Herein, we tie the release of pneumolysin to the biofilm phenotype and demonstrate that heart resident macrophages are depleted in a pneumolysin dependent manner. Rapid macrophage death then precludes immune cell infiltration by restricting cytokine and chemokine production. Thus, this study changes our understanding of how biofilms modulate the host response and corrects our prior report by providing a more detailed molecular explanation for the observed immunoquiescent phenotype.

Our cardiac findings are consistent with the known role for Ply in establishing early residency within nasopharynx [50]. They also explain why TEM imaging of nasal septa from colonized mice showed considerably greater mucosal epithelial cell perturbation, yet less cytokine production when compared to mice colonized with a biofilm deficient mutant [15]. Although cardiomyocytes are killed as a result of pneumolysin exposure [6, 8], and the same occurs for respiratory epithelial cells [51], the fact that these cells are not immune cells may explain why their damage or death does not lead to overt cytokine and chemokine production [15]. We therefore propose that biofilm-mediated release of pneumolysin and resident macrophage killing may be an explanation for the low levels of cytokines and chemokines detected during nasopharyngeal colonization. This notion warrants testing.

It is important to note that mice infected with strain 6A-10 did not develop cardiac microlesions and instead had pneumococci entrapped within cardiac macrophages. This indicates not all clinical isolates are capable of causing microlesions as seen with TIGR4. The most likely explanation for this difference is the considerable genetic heterogeneity that occurs between different clinical isolates; ~10% of their genomes [24]. For example, 6A-10 does not encode RD12. In fact, we identified RD12 in only five of the *S*. *pneumoniae* genomes publically available through PubMed. While the association between severe *S*. *pneumoniae* disease, cardiac damage, and adverse cardiac events in humans is now unequivocal [1, 2, 4–6, 9], the exact cardio-pathological hallmarks and how they vary between clinical isolates remains unknown. These are undoubtedly impacted by the genetic content of the invading strain, and based upon our results herein, the resultant biofilm forming ability and strain-specific dynamics of pneumolysin release. Importantly, the role RD12 plays could presumably be compensated for by the other bacteriocin systems known to be present in *S*. *pneumoniae* [52].

One striking observation was the anatomical-site specific differences in pneumococcal virulence gene expression. For example, the genes encoding Ply and PavB were expressed in the heart but not the blood. Similarly striking were the major differences between *in vivo* and *in vitro* gene expression, which shows that our *in vitro* condition is not an adequate mimic of the host. As such, a pressing need to expand on our studies and comprehensively determine the transcriptome of *S*. *pneumoniae* in the lungs, nasopharynx, and other body sites is now highly evident. Such a comprehensive *in vivo* gene atlas would not only elucidate the pathogenic process but would also serve to identify pneumococcal proteins that are consistently expressed across body sites. Presumably, these would be among the most suitable vaccine antigens to protect against all stages of pneumococcal disease. For example, that antibody against Ply does not protect against bacteremia following TIGR4 challenge was shown herein following passive immunization and also reported as part of our original manuscript that describes cardiac microlesion formation [6]. Alternatively, PspA, which was highly expressed in both heart and blood and has already been shown to be a protective antigen [53], may be such a candidate antigen.

In summary, we report that cardiac microlesion formation during IPD involves an intracellular stage and requires that the pneumococci transition into a biofilm. Pneumococci within the heart have a unique transcriptional profile that surprisingly does not include several established virulence determinants but included genes within three RDs that were drastically up-regulated *in vivo*. This highlights the dynamic nature of pneumococcal gene expression *in vivo* and this should be taken into consideration when considering vaccine antigens. We show that RD12 is important for biofilm formation *in vitro* and reduced cardio-pathology *in vivo*. This possibly implicates an important role for bacteriocin systems in human disease. Finally, we show that biofilm pneumococci subvert host response through the rapid killing of cardiac macrophages in a Ply-dependent manner. Moving forward, studies that examine *in vivo* bacterial gene expression are critical for a fuller understanding of bacterial pathogenesis, host-pathogen interactions and rational vaccine design.

## Materials and methods

### Bacterial strains

*S*. *pneumoniae* serotype 4, strain TIGR4 was the parent wildtype strain used and its annotated finished (gap-free) genome is available [54]. An isogenic mutant lacking *spxB* (T4 Δ*spxB*) was created by allelic exchange using a mutagenic PCR construct consisting of the *ermB* erythromycin cassette flanked by upstream and downstream fragments of the gene as previously described [25]. Isogenic TIGR4 mutants lacking the Regions of Diversity, RD2 (T4ΩRD2), and RD12 (T4ΩRD12) have been previously described [25]. *S*. *pneumoniae* serotype 6A, strain 6A-10 and its isogenic mutant lacking *ply* (6A-10Δ*ply*) have also been previously described [15]. Pneumococci were grown in Todd Hewitt Broth (THB) (Acumedia, Neogen) with 0.5% yeast extract (THY) at 37°C in 5% CO_2_ for experiments.

### Ethics statement

All mouse experiments were reviewed and approved by the Institutional Animal Care and Use Committees at The University of Alabama at Birmingham, UAB (Protocol # IACUC-20175) and The University of Texas Health San Antonio (Protocol # 13032-34-01C). At both institutes animal care and experimental protocols adhered to Public Law 89–544 (Animal Welfare Act) and its amendments, Public Health Services guidelines, and the Guide for the care and use of Laboratory Animals (U.S. Department of Health & Human Services).

### Infection of mice

Female 6-7-week-old BALB/cJ mice were challenged with ~10^3^ CFU of exponential phase pneumococci in 100μL phosphate-buffered saline (PBS) by intraperitoneal injection. For studies with Blood Isolated Pneumococci (BIP)- and Heart Isolated Pneumococci (HIP)-infected mice, pneumococci were obtained as described below. Blood for assessment of bacterial burden was obtained by tail bleeds. At fixed time points or when deemed moribund, mice were euthanized by CO_2_ asphyxiation and death was confirmed by pneumothorax before heart-collection. For passive immunization, mice were administered 3μg of anti-pneumolysin neutralizing antibody, PLY-4 (# ab71810, Abcam) intraperitoneally in 100μL PBS 1 hour before bacterial challenge and 14 hours post- infection.

### Transmission electron microscopy

Excised hearts were washed with ice cold PBS, fixed with phosphate buffered 4% formaldehyde with 1% glutaraldehyde, and then processed as previously described [55]. Once embedded within resin and sectioned at 1μm in thickness, electron microscopy was performed using a JEOL JEM-1230 transmission electron microscope (Peabody, MA).

### Isolation of blood- (BIP) and heart-isolated pneumococci (HIP)

Blood was collected from anesthetized mice retro-orbitally and transferred to heparin-coated collection tubes. Following euthanasia, hearts were surgically excised and washed in PBS to remove blood. Isolated hearts were homogenized in 5mL of PBS followed by filtration of the homogenate through a 40μm cell strainer. Paired blood (BIP) and strained heart (HIP) samples were flash-frozen at -80°C in working aliquots with 10% glycerol.

### Phase variant assessment

Freshly collected BIP and HIP samples were serially diluted in PBS and plated on tryptic soy agar plates supplemented with 100μL of catalase (MP Biomedicals, LLC), and incubated at 37°C in 5% CO_2_ for 16 hours. Colonies were examined under oblique transmitted light to determine the frequency of transparent versus opaque colony variant [56]. Our TIGR4 parent wildtype strain was composed of 84% transparent pneumococci and 16% opaque pneumococci.

### Antimicrobial tolerance assay for HIP and BIP

The effect of antimicrobial agents on BIP and HIP was determined using a modified version of the standard micro-dilution assay [19, 57]. These assays were conducted using samples immediately frozen after their collection to preserve their BIP or HIP phenotypes. Thawed aliquots of BIP and HIP were diluted in Dulbecco’s Modified Eagle’s Medium, DMEM (Corning) containing penicillin (0.125μg/mL) or erythromycin (0.5μg/mL) to a final concentration of 10^3^ CFU/mL. BIP and HIP in DMEM without antibiotic were used as controls. At regular time intervals of 1 hour, 5μL of each bacterial suspension was spotted on tryptic soy blood agar plates (Remel, USA) and incubated at 37°C in 5%CO_2_ for 16 hours. The percentage fraction of antibiotic tolerant pneumococci in each sample per time point was calculated as: (# recovered CFU in antibiotic / # CFU in non-antibiotic control) x 100.

### Immunofluorescent and lectin staining of cardiac sections

Hearts collected from infected mice were washed thoroughly with PBS then embedded in cassettes with Optimal Cutting Temperature Compound (Tissue-Tek, 4583). Frozen 7μm thick cardiac sections were fixed with 10% neutral buffered formalin, permeabilized in 0.2% Triton X and blocked with PBS containing 5% serum from species to which the secondary antibody belonged (blocking buffer). Sections were then incubated overnight at 4°C with blocking buffer containing a 1:1000 dilution of primary antibody: rabbit anti-serotype 4 capsular polysaccharide antibody (Statens serum Institut: cat #16747), or rabbit anti-mH2A.1 antibody (EMD Millipore, cat#ABE215). The next day sections were vigorously washed with 0.2%Triton X and then incubated for 1 hour at room temperature (RT) with blocking buffer containing secondary antibody at 1:2000 dilution: FITC labeled goat α-rabbit antibody (Jackson Immuno Research, cat#111-096-144), or rhodamine labeled donkey α-rabbit antibody (EMD Millipore, cat#AP182R). For neutrophil and macrophage staining, cardiac sections were incubated with rat α-mouse Ly-6G primary antibody (clone 1A8; BD Biosciences) and rat α-mouse CD107b primary antibody (clone M3/84; BD Biosciences) diluted at 1:500 in the blocking buffer for 1 hour at RT. After incubation, sections were washed and incubated with Alexa 488- conjugated goat α-rat secondary antibody (Jackson Immuno Research, West Grove, PA) diluted at 1:500 in blocking buffer for 45 minutes at RT. Exposed galactose was stained for with fluorescein labeled *Erythrina crystagalli* lectin (Vector Laboratories, FL-1141) after blocking with carbo-free blocking solution for 1 hour (Vector Laboratories, SP-5040). All slides were stained with DAPI (Molecular Probes by Life Technologies, R37606) mounting the sections with FluorSave (Calbiochem: 345789) and covering with coverslip for visualization. Images of cardiac sections were captured at The University of Texas Health San Antonio using a Zeiss LSM 710 confocal microscope and at UAB using a Leica LMD6 microscope equipped with DFC3000G monochrome camera. Image stitching of whole IFM stained cardiac sections was performed using the Leica LASX software. When indicated, cardiac microlesions were enumerated by counting foci of pneumococci in three capsule stained heart sections, each section at least >50μm apart. The first section was cut 300 μm into the heart from the surface.

### Static biofilm formation assays

Static pneumococcal biofilms were grown in 6-well polystyrene plates (COSTAR) as previously described [15]. FilmTracer LIVE/DEAD Biofilm viability kit (Invitrogen, L10316) was used to determine viability of 24-hour biofilms grown on 1% BSA coated coverslips as per Manufacturer’s protocols. Biofilms were visualized using a Leica LMD6 microscope with DFC3000G monochrome camera and Z-stacked to construct 3D-images.

### Adhesion assays

Adhesion assays on HL-1 mouse atrial cardiomyocytes (generously provided by Dr. William Claycomb, New Orleans, LA) and RBCEC6 rat brain capillary endothelial cells were performed as described previously [58]. All experiments were performed in triplicates.

### RNA isolation

For HIP-RNA, hearts were excised from HIP-infected mice (n = 3) when deemed moribund. Hearts were rinsed, diced, fragments washed with ice cold PBS, and the fragments homogenized in RNAprotect bacteria reagent (Qiagen) and stored at -80°C. We empirically determined that high titers of pneumococci were necessary to capture the bacterial transcriptome in the bloodstream when using dual RNA-seq. These levels were not routinely seen following conventional challenge and we resorted to neutrophil depletion to achieve the necessary titers (>10^7^ CFU/mL). Ten mice pre-depleted for neutrophils using anti-Ly6G antibody (BioXCell, clone RB6-8C5) were infected with TIGR4. Blood was collected in RNAprotect bacteria reagent (Qiagen) when the mice were deemed moribund such that blood from 5 mice were pooled as one sample (n = 2 BIP samples) and stored at -80°C. On the day of total RNA isolation from heart homogenates, the samples were thawed and spun down to discard the supernatant. The pellets were further homogenized in 600 μL RLT with B-ME buffer using a motorized mortar for 30 seconds. The re-homogenized samples were then disaggregated in a Qiashredder followed by RNA extraction with the RNeasy Micro Kit (Qiagen) with DNase treatment on column and in solution. The isolated RNA was quantitated using Nanodrop and Bioanalyzer. Samples were then depleted of rRNAs using the Ribo-Zero rRNA Removal Kit for Gram-positive bacteria and human/mouse/rat (Illumina, San Diego, CA).

For the *in vitro* biofilm and planktonic samples: planktonic mid-log phase (OD_620nm_ = 0.5) TIGR4 grown in THB were used to seed continuous once-flow through biofilm reactors. Biofilms were allowed to grow for 48 hours prior to collection of bacteria. RNA was isolated from the paired planktonic seed cultures (n = 3) and their respective biofilms (n = 3). Total RNA was extracted from each replicate separately using enzymatic lysis of pneumococcal cells (10μL mutanolysin at 25 units/μL, 20μL proteinase K at 20mg/mL, 15μL lysozyme AT 15mg/mL, and 55μL TE) followed by RNA extraction using the same protocol mentioned above. Blood samples were isolated in a similar manner as biofilm and planktonic samples, including enzymatic lysis, except that cells were first disaggregated with the Qiashredder like for the heart samples.

### RNA-seq and comparative transcriptomic analyses

Illumina strand-specific RNA-seq libraries were constructed with the TruSeq RNA Sample Prep kit (Illumina, San Diego, CA) per manufacturer’s protocol. Between 1^st^ and 2^nd^-strand cDNA synthesis, the primers and nucleotides were removed from the samples with NucAway spin columns (Ambion, Austin, TX). The 2^nd^ strand was synthesized with a dNTP mix containing dUTP. Adapters containing 6 nucleotide indexes were ligated to the double-stranded cDNA. After adapter ligation, the 2^nd^ strand cDNA was digested with 2 units of Uracil-N-Glycosylase (Applied Biosystems, Carlsbad, CA). Size selection of the library was performed with AMPure XT beads (Beckman Coulter Genomics, Danvers, MA). In order to achieve sufficient levels of RNA sequencing for bacterial transcripts in the presence of an abundance of mouse transcripts (dual RNA-seq), libraries were loaded on 150nt paired-end runs of the Illumina HiSeq4000 sequencing platform as follows: half of a channel for each of the BIP and HIP libraries, a quarter of a channel for each of the biofilm and planktonic libraries, and an eighth of a channel for each of the three-control uninfected mouse heart libraries. Raw RNA-seq data and associated *in silico* analyses for BIP, HIP, biofilm and planktonic TIGR4 are deposited at GEO (accession number GSE86118). Reads from each of the 3 HIP-infected heart samples, 2 pooled BIP samples, 3 biofilm- and 3 planktonic- pneumococci samples were mapped onto the reference *S*. *pneumoniae* TIGR4 genome using Bowtie version 0.12.9[59]. The alignment BAM files from Bowtie were used to compute gene expression levels and test each gene for differential expression. The number of reads that mapped to each TIGR4 gene was calculated using the python package HTSeq version 0.4.7 [59]. Differential gene expression analysis was conducted using the DESeq R package version 1.5.24 (available from Bioconductor)[60]. The DESeq analysis resulted in the determination of potential differentially expressed genes when compared between the control planktonic samples and the *in vitro* biofilm, heart and blood samples, respectively. Read counts for each sample were normalized for sequencing depth (RPKM) and distortion caused by highly differentially expressed genes. Then the negative binomial (NB) model was used to test the significance of differential expression between three pairs of conditions (i.e. *in vitro* biofilm vs. planktonic, heart vs. planktonic, and blood vs. planktonic). The differentially expressed genes were deemed significant if the False Discovery Rate (FDR) was less than 0.05, the gene expression was above the 10^th^ percentile and showed greater than 2-fold change difference (up-regulated or down-regulated) between the paired conditions.

### Principal component analysis (PCA)

We performed PCA analysis (R statistical software v2.15.2) on log_10_(RPKM) gene expression values across all growth conditions and all replicates for each condition, i.e. *in vitro* planktonic (n = 3), *in vitro* biofilm (n = 3), heart (n = 3) and blood (n = 2) samples. This analysis was heavily skewed by genes that showed no expression at all *in vivo* (RPKM = 0). In order to circumvent this problem, we used a cutoff of RPKM>1 across all conditions prior to PCA analysis. This analysis revealed tight clustering of all replicates within a given condition. In addition, principal component 1 (PC1, **Fig 5A** X-axis) separated *in vivo* from *in vitro* conditions, while PC2 (**Fig 5A** Y-axis) separated biofilm (*in vitro* and heart) from planktonic (*in vitro* and blood). It should be noted that the overall depth of coverage of gene expression value interrogation in the *in vivo* conditions was significantly lower than for *in vitro* cultures (due to the overwhelming presence of mouse transcripts), explaining the PCA skew when including genes with zero expression. Yet, the distribution of RPKM values across the TIGR4 genome revealed similar average RPKMs of 605 and 520 for *in vivo* and *in vitro* samples, respectively. In addition, maximum expression values observed were ~25,000 *in vitro* in contrast to ~125,000 *in vivo*. Finally, many of the very highly *in vivo* expressed genes were part of operons within the RDs described in the results and discussion sections. Therefore, our interrogation of the *in vivo* gene expression profiles was robust. We then computed correlation values (R statistical software v2.15.2) for all genes to PC1 and PC2 separately. Using a correlation cutoff of 85%, we identified 142 and 105 genes that correlated with PC1 and PC2, respectively (**S8 and S9 Figs**).

### Circos plot

A Circos plot for whole transcriptome comparisons of BIP, HIP, *in vitro* biofilm- and *in vitro* planktonic-pneumococci samples for expression levels of 2,105 TIGR4 genes was generated. Read counts were computed for 2,105 genes within each sample using the python package ‘htseq’. Genes with a count of 0 across all samples were excluded resulting in 2090 genes. The read counts were normalized for library size in each sample and a normalized value of counts per million-mapped-reads (CPM) was computed for all genes. Additionally, genes with CPM values < 1 in all samples were excluded resulting in 1969 genes. The mean expression value for each gene was computed within each of the 4 conditions. The average gene expression values were converted to z-scores and were used to rank the genes within each condition. Genes with z-scores ≥ +1 were classified as genes with ‘high’ expression (red color) while genes with z-scores ≤ -1 were classified as genes with ‘low’ expression (green color). The remaining genes were classified as genes with ‘intermediate’ expression. These gene expression values, gene ranks and gene stratification were utilized to generate the circular plots (**Fig 5B**) using the ‘Circos’ tool version 0.69 [61].

### qRT-PCR analyses

qRT-PCR confirmation of RNA-seq results was conducted using the ABI 7900HT Fast Real-Time PCR System (Applied Biosystems). 69 pneumococcal genes of interest were analyzed by qRT-PCR across all conditions and replicates of the RNA samples used for RNA-seq. Gene expression data was normalized using three genes that were unregulated across all conditions: SP_0378, SP_1489, and SP_1667. The comparative critical threshold (Ct) method was used to determine the ΔCt. The ΔCt was then correlated to the log_2_(Ratio) of expression from RNA-seq results through a linear regression. The primer sequences used for qRT-PCR are provided in **S3 Table**.

### Flow cytometric analyses

Freshly excised hearts were minced on ice and digested in serum free Iscoves DMEM supplemented with 2mg/mL of Collagenase Type 2 (Worthington, Cat #LS004176) and 0.02mg/mL of Deoxyribonuclease I from bovine pancreas (Sigma, Cat #DN25-100MG) at 37°C for 30 minutes. These cell suspensions were then neutralized with IDMEM containing 10% FBS and filtered through 0.45μm strainer before blocking with 2.4G2 (BD Pharmingen, Cat #553141) for 30 minutes on ice, and staining with Gr-1-APC (Clone RB6-8C5, eBioscience), CD11b-APC-Cy7 (M1/70, BD Pharmingen), Ly 6C-PerCP-Cy5.5 (HK1.4, eBioscience), Ly6G-FITC (1A8, eBioscience), MHC-II PE-Cy5 (M5/114.15.2, eBioscience), MerTK-PE (DS5MMER, eBIoscience), F4/80-PE-Cy5 (BM8, eBioscience) and CD64-Biotin (X54-5/7.1, Biolegend and used in conjunction with Streptavidin PE-Cy7, eBioscience) antibodies for 30 minutes on ice protected from light. Cells were washed with PBS prior to flow cytometry. The samples were then analyzed on BD LSR-II (UAB Flow Cytometry Core Facility). Neutrophils were identified as Gr-1^+^CD11b^+^Ly-6G^+^F4/80^-^MHC-II^-^. Cardiac macrophages were first gated on F4/80^+^CD11b^+^ cells and further gated for expression of MerTK and CD64. Flow cytometry data were analyzed using FlowJo software. Percent neutrophils and macrophages were determined as (Percent live gated cells from the heart) x (percent positive).

### *In vitro* cell infection, LDH release cytotoxicity assays, and cytokine analysis

Percentage cytotoxicity and TNFα, CXCL1, and CXCL2 production by mouse J774A.1 macrophages and HL-1 atrial cardiomyocytes at designated time-points following exposure to an equal biomass of pneumococci (corresponding to multiplicity of infection of ~10 planktonic bacteria in DMEM) were measured by Pierce LDH cytotoxicity assay kit (Thermo Scientific) and ELISA (R&D systems), respectively. To set equal biomass, biofilm-pneumococci were flushed out of the continuous flow-through systems using THY and the optical density of the biofilm suspension adjusted to that of log-phase planktonic bacteria grown in parallel in THY (OD_620nm_ = 0.5).

### Western blot analysis

Samples for western blot quantification for pneumolysin levels were prepared by lysing pellets of planktonic- and flow through biofilm-cultures of *S*. *pneumoniae* at equal biomass using pneumococcal lysis buffer (0.01% SDS, 0.1% DOC, and 0.015 M Na-citrate) and concentrating supernatant proteins using acetone precipitation [62]. Samples were frozen with protease inhibitors (Sigma). Equal biomass of pellets and supernatants were loaded after BCA quantification. Isogenic pneumolysin deficient strains were tested as the negative controls. Normalized densitometric quantification of pneumolysin levels in the supernatant was performed using ImageJ processing software.

### Statistics

Statistical analysis of *in silico* data is provided in the Supplemental Experimental Procedures. For wet lab research, multiple group analyses were performed using One-Way ANOVA Kruskall-Wallis Test with Dunn’s Multiple Comparison Post-test. For all non-parametric data sets, we used a Mann-Whitney test while Student’s *t*-test was used to analyze parametric data sets. These statistical analyses were performed using Prism 5.0 (GraphPad Software: La Jolla, CA). Data are represented as mean ± SEM. *P*-value ≤0.05 were deemed significant.

## Supporting information

S1 Fig *S*. *pneumoniae* forms biofilms within cardiac microlesions. **(A) Increasing size of cardiac microlesions was associated with an accumulation of ghost pneumococci.** Percentage of non-electron dense (i.e. ghost) pneumococci found within cardiac microlesions of different sizes. The number of ghost pneumococci per cardiac microlesion was determined manually using a gray-scale density cutoff such that cells that had electron density <30% in the captured TEM images were considered ghosts. At least 3 cardiac microlesions per designated size were examined from hearts of 12 infected mice. Statistical analyses were performed using non-parametric one-way ANOVA (Kruskal-Wallis test) with Dunn’s multiple comparison test. *P* value: * ≤ 0.05, ** ≤ 0.01; data is represented as mean ± SEM.
(PDF)

S2 Fig Serotype 6A, strain 6A-10 is capable of translocating into the heart but incapable of forming form cardiac microlesions. **(A)** Representative immunofluorescent stained images of cardiac sections from mice infected with 6A-10, 30 hours post infection (n = 3 mice). Cardiac sections were stained using serotype 6A capsule polysaccharide antisera (*green*) and DAPI (*blue*). Individual pneumococci dispersed within the myocardium are shown using arrows. **Inset,** High power immunofluorescent image of the marked section depicting pneumococci around vasculature (white dotted lines). **(B)** Representative transmission electron microscopy (TEM) images of cardiac sections from BALB/cJ mice infected with *S*. *pneumoniae* strain 6A-10 30 hours post-infection (n = 4). TEM imaging of 6A-10 infected hearts showed pneumococci (black arrows) within cardiac macrophages (black solid line) adjacent to the vasculature (white solid line). The black dotted lines depict the macrophage nucleus while the white dotted line depicts an erythrocyte within the vasculature. The black dashed line shows the cardiomyocyte nucleus.
(PDF)

S3 Fig Specificity of immunofluorescent probes used to detect different components of pneumococcal biofilms within microlesions. **(A)** Isotype control (ITC) staining for **(A.1)** rabbit anti- serotype 4 capsule specific antisera and for **(A.2)** rabbit anti-mH2A.1 histone anti-sera using normal rabbit sera.
(PDF)

S4 Fig T4 *ΔspxB* is not attenuated for bacteremia or adhesion to vascular endothelial cells. **(A)** Bacterial titers in the blood of mice infected with TIGR4 (T4) and the isogenic *spxB* deficient mutant (T4 Δ*spxB*) post-infection. Statistical analysis was performed using Mann-Whitney test. **(B)** The adhesive ability of T4 *ΔspxB* to rat brain capillary endothelial cells (RBCEC6) was determined *in vitro*. Values are expressed as fold-increase in adhesion of T4 *ΔspxB* relative to the wild-type TIGR4 strain (T4). Statistical analysis was performed using Mann-Whitney test. Data is represented as mean ± SEM. No statistically significant difference was observed. *P* value: ** ≤ 0.01; data are represented as mean ± SEM.
(PDF)

S5 Fig Exposure of terminal galactose in areas surrounding microlesions is pneumococcus mediated. Representative tile-stitched image of whole heart sections from uninfected control mice (n = 3), TIGR4 infected mice (n = 3), myocardial infarcted mice (n = 1) and sham surgery mice (n = 1). The cardiac sections (stained with DAPI, *blue*) were probed for TIGR4 (*red*), using serotype 4 capsule polysaccharide antisera, and for exposed galactose residues (*green*), using fluorescein labeled *Erythrina crystagalli* lectin, within the heart. Sterile tissue injury due to experimentally induced myocardial infarction did not result in galactose exposure as observed during TIGR4- mediated microlesion formation.
(PDF)

S6 Fig Heart- and blood- isolated pneumococci have comparable adhesive and invasive abilities towards endothelial cells. Adhesion and invasion of heart isolated pneumococci, HIP (n = 4) compared to blood isolated pneumococci, BIP (n = 4) to rat brain capillary endothelial cells (RBCEC6) *in vitro*. Values are expressed as fold-increase in HIP relative to BIP. Experiments were done using 4 sets of paired HIP and BIP samples collected from 4 individual mice (i.e. 4 biological replicates). Each sample pair was tested against each other using 3 technical replicates on each cell line. The average of each set of technical replicates, was used to create the figure panel and for statistical analysis. Statistical analysis was performed using Mann-Whitney test. No statistically significant difference was observed; data are represented as mean ± SEM.
(PDF)

S7 Fig Validation of RNA-seq expression data. Gene expression fold change levels for (**A)** *in vitro* biofilms, **(B)** Heart- isolated pneumococci (HIP), and **(C)** blood Isolated pneumococci (BIP) each in comparison with planktonic pneumococci as determined using RNA-Seq analysis were confirmed using qRT-PCR for 69 candidate genes. Expression fold change values obtained by RNA-seq analysis (X-axis) and qRT-PCR (Y-axis) are in good agreement as evidenced from strong correlation coefficients. The primer sequences used for qRT-PCR are provided is S3 Table.
(PDF)

S8 Fig Heat map of gene expression levels for genes correlated with PC2. Heat map depiction of log_10_(RPKM) gene expressions levels for genes that drive the separation of planktonic (*in vitro* and BIP) and biofilm (*in vitro and* HIP) populations along the PC2 Y-axis of **Fig 5A**. 105 genes correlated at ≥85% with PC2.
(PDF)

S9 Fig Heat map of gene expression levels for genes correlated with PC1. Heat map depiction of log_10_(RPKM) gene expressions levels for genes that drive the separation of *in vitro* (biofilm and planktonic) and *in vivo* (HIP and BIP) populations along the PC1 X-axis of **Fig 5A**. 142 genes correlated at ≥85% with PC1. Note that genes within RD2, RD6 and RD12 were among the major PC1-correlated genes.
(PDF)

S10 Fig **(A) Organization of genes within RD12.** Putative gene names and predicted functions are shown. **(B) T4ΩRD12 exhibits no long-term growth defects.** Growth curves of TIGR4, T4ΩRD2, and T4ΩRD12 in Todd Hewitt Broth with 0.5% yeast extract (THY) are presented. Despite a delayed log phase growth T4ΩRD12 showed no long-term growth defects. Experiments were performed in triplicates. **(C) T4ΩRD12 undergoes normal autolysis during bile solubility assay.** Comparative autolytic properties of TIGR4, ΩRD2, ΩRD12 and Δ*lytA* (negative control) isogenic mutants on treatment with pneumococcal lysis buffer (0.01% SDS, 0.1% DOC, AND 0.015 M Na-citrate) are shown. The *lytA* deletion mutant was a kind gift from Dr. Terry Brissac, *personal communication*.
(PDF)

S11 Fig Deletion of RD12 had no impact on capsule levels. Normalized densitometric quantification of capsule levels by expressed by equal biomass of planktonic wildtype TIGR4 (n = 3), planktonic T4ΩRD12 (ΩRD12) (n = 3) is provided. An isogenic capsule deficient TIGR4 strain (T4R) was tested as the negative control. Statistical analysis was performed by comparison of capsule levels from planktonic T4ΩRD12 and planktonic T4R to planktonic- wildtype TIGR4 (n = 3) using One-way ANOVA. Representative Immunodot blot for capsule levels is shown.
(PDF)

S12 Fig Biofilm pneumococci rapidly kill macrophages in a pneumolysin- dependent manner to subvert host immune response. **(A) Macrophages and cardiomyocytes do not produce CXCL1 in response to pneumococcal challenge.** CXCL1 cytokine production by J774A.1 macrophages and HL-1 cardiomyocytes following 4 hour exposure to equal biomass of planktonic- and biofilm- TIGR4 (T4) or the RD12 deficient mutant strain. N.D. denotes not detectable. Statistical analysis was performed using non-parametric One-way ANOVA (Kruskal-Wallis Test); no significant CXCL1 production was observed compared to untreated cells. **(B) Biofilm pneumococci mute the macrophage CXCL2 response in a pneumolysin-dependent manner.** CXCL2 production by J774A.1 macrophages at designated time points following exposure to equal biomass of planktonic-, biofilm- TIGR4 (T4), planktonic-, biofilm- T4 Δ*ply* and planktonic-, biofilm- T4 Δ*ply* complemented with exogenous recombinant pneumolysin (rPLY) at a concentration 0.3μg/mL. Experiments were performed as three biological replicates with 3 technical replicates each. Statistical analysis was performed using ordinary one-way ANOVA. **(C) Pneumolysin is not required for biofilm formation.** Static biofilm-forming ability of TIGR4 (T4) and it isogenic pneumolysin deficient mutant (T4 Δ*ply*) was assessed in a 48-hour 6-well polystyrene plate model (n = 5 experiments). Biofilm biomass was measured using crystal violet staining. Statistical analysis was performed using Student’s *t*-test. No statistically significant difference was observed. Representative crystal violet stained biofilms are shown. Data are represented as mean ± SEM.
(PDF)

S13 Fig 6A-10 biofilms do not show enhanced release of pneumolysin and have a comparatively modest capability to kill macrophages and subvert cytokine production as a biofilm. **(A)** Western blots for pneumolysin levels in equal biomass of whole cell lysates (pellets) and supernatants of planktonic- wildtype 6A-10 (n = 3), and biofilm- wildtype 6A-10 (n = 3). An isogenic pneumolysin deficient 6A-10 strain (6A-10 Δ*ply*) was tested as the negative control. **(B)** LDH release cytotoxicity assay of J774A.1 macrophages challenged with equal biomass of planktonic-, biofilm- 6A-10, planktonic-, biofilm- 6A-10 Δ*ply* and planktonic-, biofilm- 6A-10 Δ*ply* complemented with exogenous recombinant pneumolysin (rPLY, 0.3μg/mL) as determined at 0, 1, 2, 4 hours post-infection (n = 3 biological replicates, each with 3 technical replicates). Statistical analysis was performed using ordinary one-way ANOVA. **(C)** TNFα production by J774A.1 macrophages at designated time points following exposure to an equal biomass of planktonic-, biofilm- 6A-10, planktonic-, biofilm- 6A-10 Δ*ply* and planktonic-, biofilm- 6A-10 Δ*ply* complemented with exogenous recombinant pneumolysin (rPLY, 0.3μg/mL) as determined at 0, 1, 2, 4 hours post-infection (n = 3 biological replicates, each with 3 technical replicates). Statistical analysis was performed using ordinary one-way ANOVA. **(D)** CXCL2 production by J774A.1 macrophages at designated time points following exposure to an equal biomass of planktonic-, biofilm- 6A-10, planktonic-, biofilm- 6A-10 Δ*ply* and planktonic-, biofilm- 6A-10 Δ*ply* complemented with exogenous recombinant pneumolysin (rPLY, 0.3μg/mL) as determined at 0, 1, 2, 4 hours post-infection (n = 3 biological replicates, each with 3 technical replicates). Statistical analysis was performed using ordinary one-way ANOVA.
(PDF)

S14 Fig Heart invaded pneumococci rapidly kill cardiac macrophages in a pneumolysin- dependent manner to subvert inflammation. **(A, B)** Pneumococcal titers in the (**A)** blood and **(B)** heart of mice infected with TIGR4 (T4) and T4 Δ*ply* 30 hours post-infection. Mann-Whitney test comparing the mutant strain titers to the wildtype TIGR4 titers was performed. **(C)** Representative high magnification immunofluorescent microscopy images of cardiac microlesions from mice infected with T4 Δ*ply* 30 hours post infection, showing presence of: capsule (stained with anti-serotype 4 capsule antibody [CPS], *red*), cardiac macrophages (stained using anti-Mac-3 antibody [Mac-3], *green*), and infiltrated neutrophils (stained with anti-Ly-6G antibody [Ly-6G], *green*). A minimum of 4 stained heart sections were examined. **(D, E)** Pneumococcal titers in the **(D)** blood and **(E)** hearts of naive and passively immunized (αPly)- mice infected with HIP or BIP 30 hours post infection. Mann-Whitney test comparing the bacterial burdens to the wildtype TIGR4 titers was performed. No statistically significant difference was observed. *P* value: ** ≤ 0.01, *** ≤ 0.001 Data are represented as mean ± SEM. **(F)** Representative high magnification immunofluorescent microscopy images of cardiac microlesions from passively immunized (αPly)- infected with BIP 30 hours post infection, showing presence of: capsule (stained with anti-serotype 4 capsule antibody [CPS], *red*), cardiac macrophages (stained using anti-Mac-3 antibody [Mac-3], *green*), and infiltrated neutrophils (stained with anti-Ly-6G antibody [Ly-6G], *green*). A minimum of 4 stained heart sections were examined.
(PDF)

S1 Table Reads-per-kilobase-per-million (RPKM) values of reads mapped to the TIGR4 genome for all genes in each replicate of each condition tested in RNA-seq. Non-sub-sampled RNA-seq RPKM gene expression values for the four experimental conditions: blood-isolated pneumococci, heart-isolated pneumococci, *in vitro* biofilm- and planktonic- TIGR4 pneumococci are listed.
(XLSX)

S2 Table Comparative transcriptomic analysis. RNA-seq gene expression log_2_ fold changes between the four experimental conditions: blood-isolated pneumococci, heart-isolated pneumococci, *in vitro* biofilm- and planktonic- TIGR4 pneumococci are listed. Normalized RNA-seq read counts (derived from reads mapped to each TIGR4 gene expressed within the TIGR4 genome) are listed. It should be noted that data from five distinct DEseq differential expression analyses are merged into a single table. In each DEseq analysis, total reads from the condition with the most reads were sub-sampled to match the number of reads of the other condition in order to increase statistical significance. As a result, normalized read count values for the same condition will vary between the different comparisons (true reads-per-kilobase-per-million [RPKM] values for all genes are presented in S1 Table). “NA” across all columns for a given analysis indicates that the gene was not included by DEseq in that comparison.
(XLSX)

S3 Table qRT-PCR primer sequences used for validation of RNA-seq data. (XLSX)
