# Supplementary figures and images for: Streptococcus pneumoniae in the heart subvert the host response through biofilm-mediated resident macrophage killing
Source: PLoS Pathog. 2017 Aug 25;13(8):e1006582. doi: 10.1371/journal.ppat.1006582 (PMC5589263; doi:10.1371/journal.ppat.1006582)

**Fig S1**

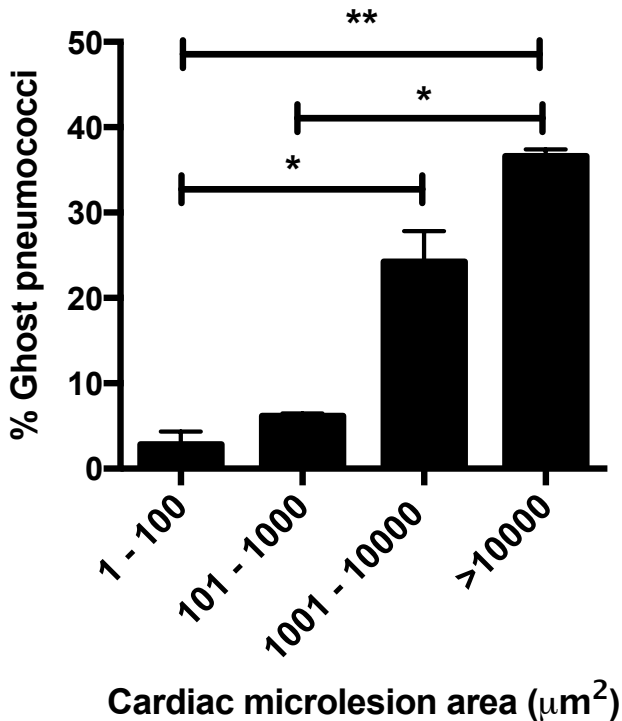

Supplement: S1 Fig — (A) Increasing size of cardiac microlesions was associated with an accumulation of ghost pneumococci. Percentage of non-electron dense (i.e. ghost) pneumococci found within cardiac microlesions of different sizes. The number of ghost pneumococci per cardiac microlesion was determined manually using a gray-scale density cutoff such that cells that had electron density <30% in the captured TEM images were considered ghosts. At least 3 cardiac microlesions per designated size were examined from hearts of 12 infected mice. Statistical analyses were performed using non-parametric one-way ANOVA (Kruskal-Wallis test) with Dunn’s multiple comparison test. P value: * ≤ 0.05, ** ≤ 0.01; data is represented as mean ± SEM. (PDF) [file ppat.1006582.s001.pdf]

**Fig S2**

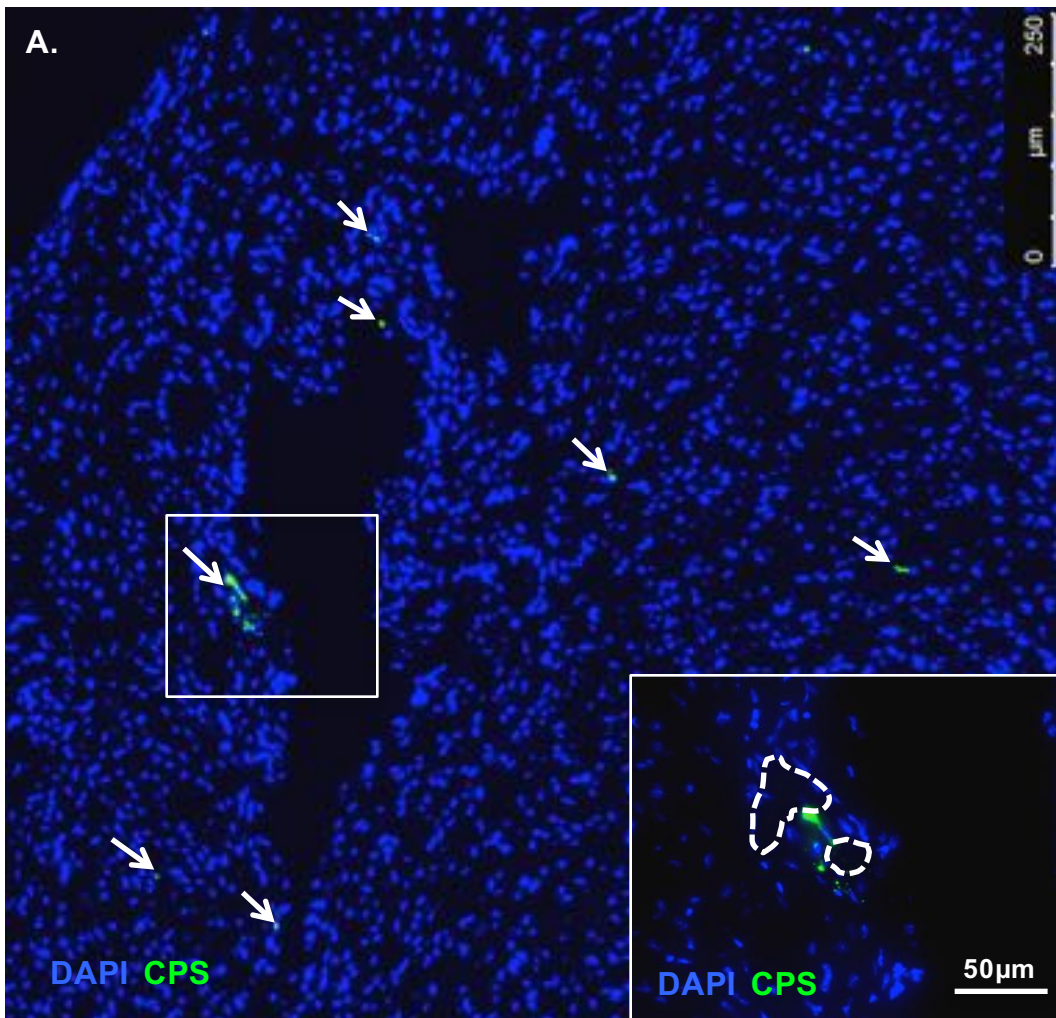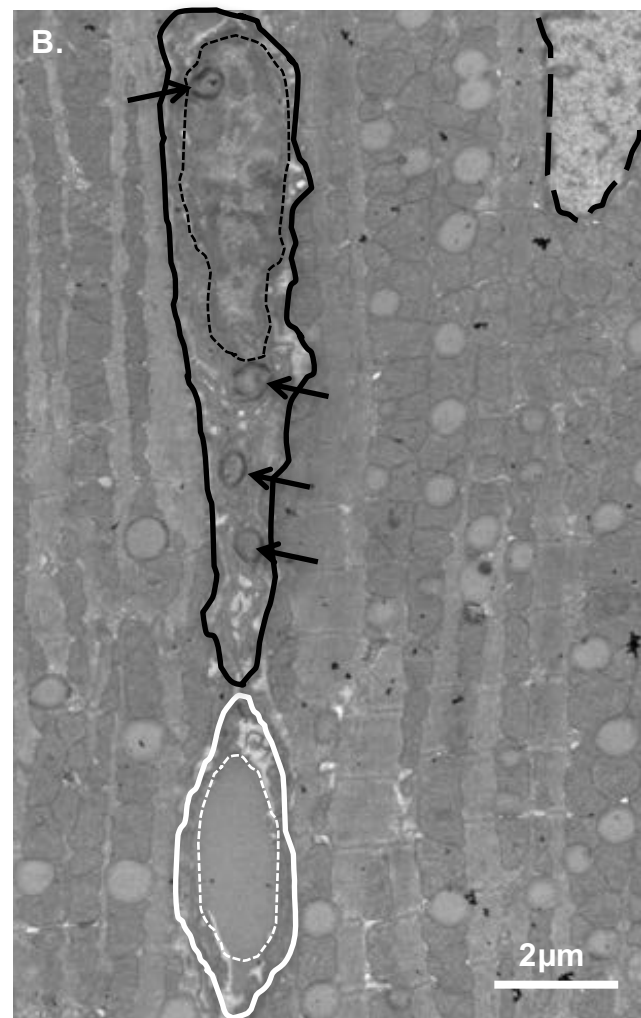

Supplement: S2 Fig — (A) Representative immunofluorescent stained images of cardiac sections from mice infected with 6A-10, 30 hours post infection (n = 3 mice). Cardiac sections were stained using serotype 6A capsule polysaccharide antisera (green) and DAPI (blue). Individual pneumococci dispersed within the myocardium are shown using arrows. Inset, High power immunofluorescent image of the marked section depicting pneumococci around vasculature (white dotted lines). (B) Representative transmission electron microscopy (TEM) images of cardiac sections from BALB/cJ mice infected with S. pneumoniae strain 6A-10 30 hours post-infection (n = 4). TEM imaging of 6A-10 infected hearts showed pneumococci (black arrows) within cardiac macrophages (black solid line) adjacent to the vasculature (white solid line). The black dotted lines depict the macrophage nucleus while the white dotted line depicts an erythrocyte within the vasculature. The black dashed line shows the cardiomyocyte nucleus. (PDF) [file ppat.1006582.s002.pdf]

**Fig S3**

Isotype Controls for microlesions components  
within Infected Heart

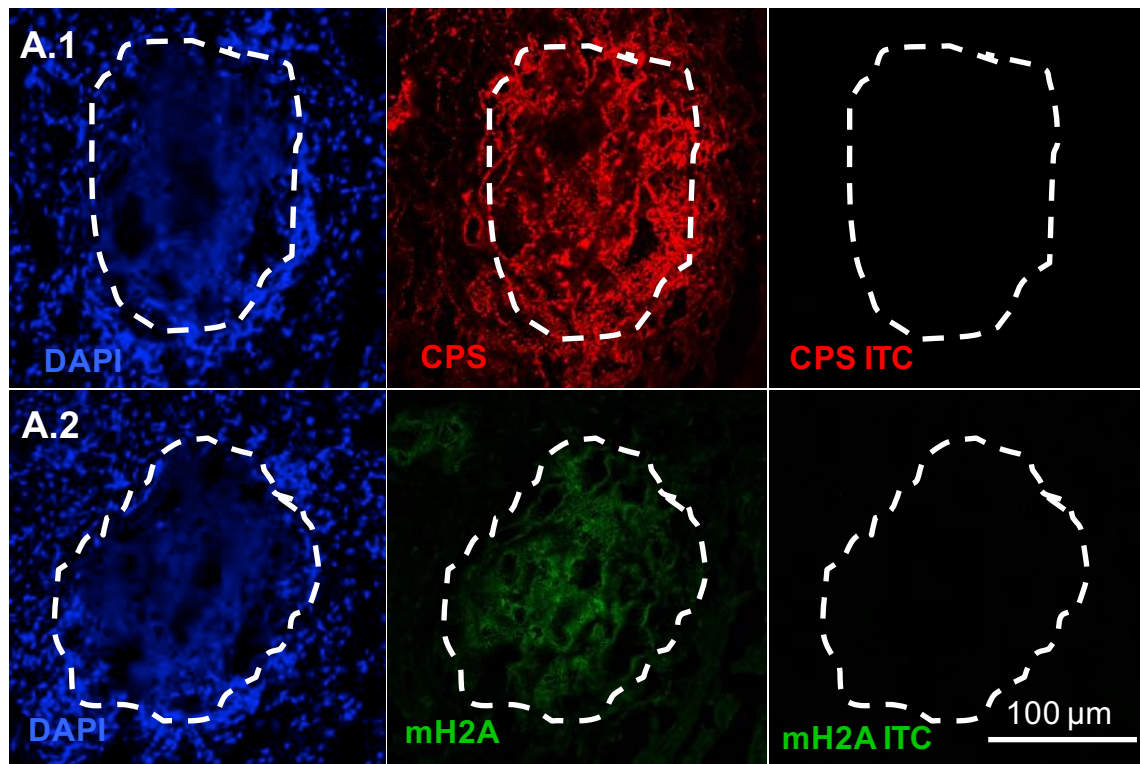

Supplement: S3 Fig — (A) Isotype control (ITC) staining for (A.1) rabbit anti- serotype 4 capsule specific antisera and for (A.2) rabbit anti-mH2A.1 histone anti-sera using normal rabbit sera. (PDF) [file ppat.1006582.s003.pdf]

**Fig S4**

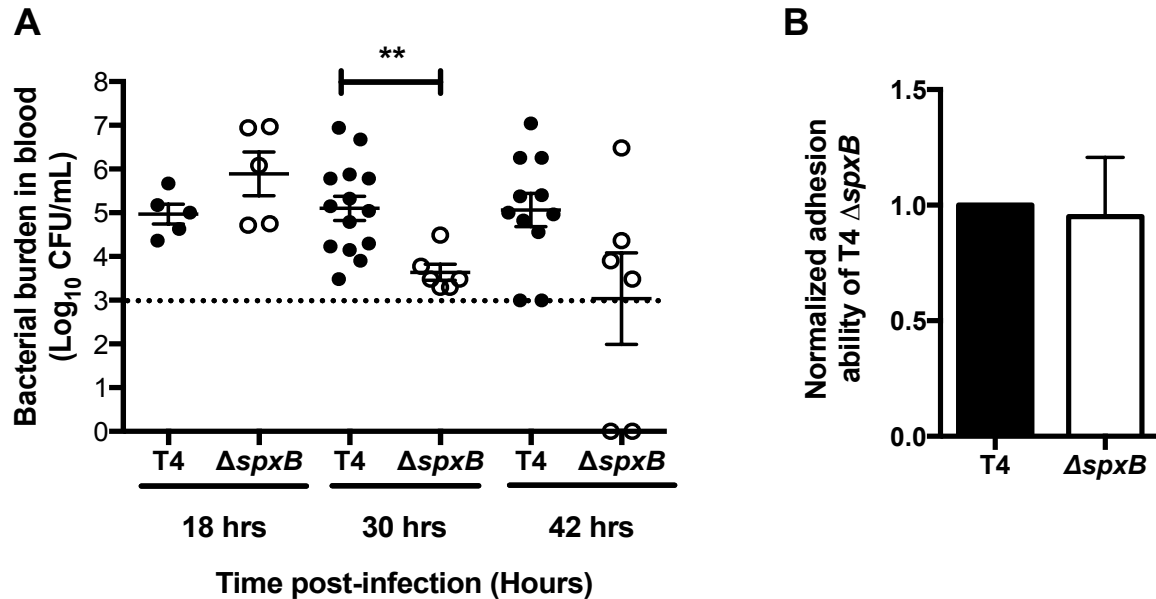

Supplement: S4 Fig — (A) Bacterial titers in the blood of mice infected with TIGR4 (T4) and the isogenic spxB deficient mutant (T4 ΔspxB) post-infection. Statistical analysis was performed using Mann-Whitney test. (B) The adhesive ability of T4 ΔspxB to rat brain capillary endothelial cells (RBCEC6) was determined in vitro. Values are expressed as fold-increase in adhesion of T4 ΔspxB relative to the wild-type TIGR4 strain (T4). Statistical analysis was performed using Mann-Whitney test. Data is represented as mean ± SEM. No statistically significant difference was observed. P value: ** ≤ 0.01; data are represented as mean ± SEM. (PDF) [file ppat.1006582.s004.pdf]

**Fig S6**

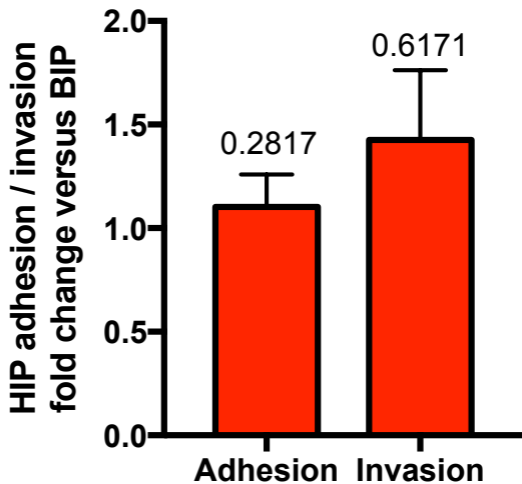

Supplement: S6 Fig — Adhesion and invasion of heart isolated pneumococci, HIP (n = 4) compared to blood isolated pneumococci, BIP (n = 4) to rat brain capillary endothelial cells (RBCEC6) in vitro. Values are expressed as fold-increase in HIP relative to BIP. Experiments were done using 4 sets of paired HIP and BIP samples collected from 4 individual mice (i.e. 4 biological replicates). Each sample pair was tested against each other using 3 technical replicates on each cell line. The average of each set of technical replicates, was used to create the figure panel and for statistical analysis. Statistical analysis was performed using Mann-Whitney test. No statistically significant difference was observed; data are represented as mean ± SEM. (PDF) [file ppat.1006582.s006.pdf]

Figure S8

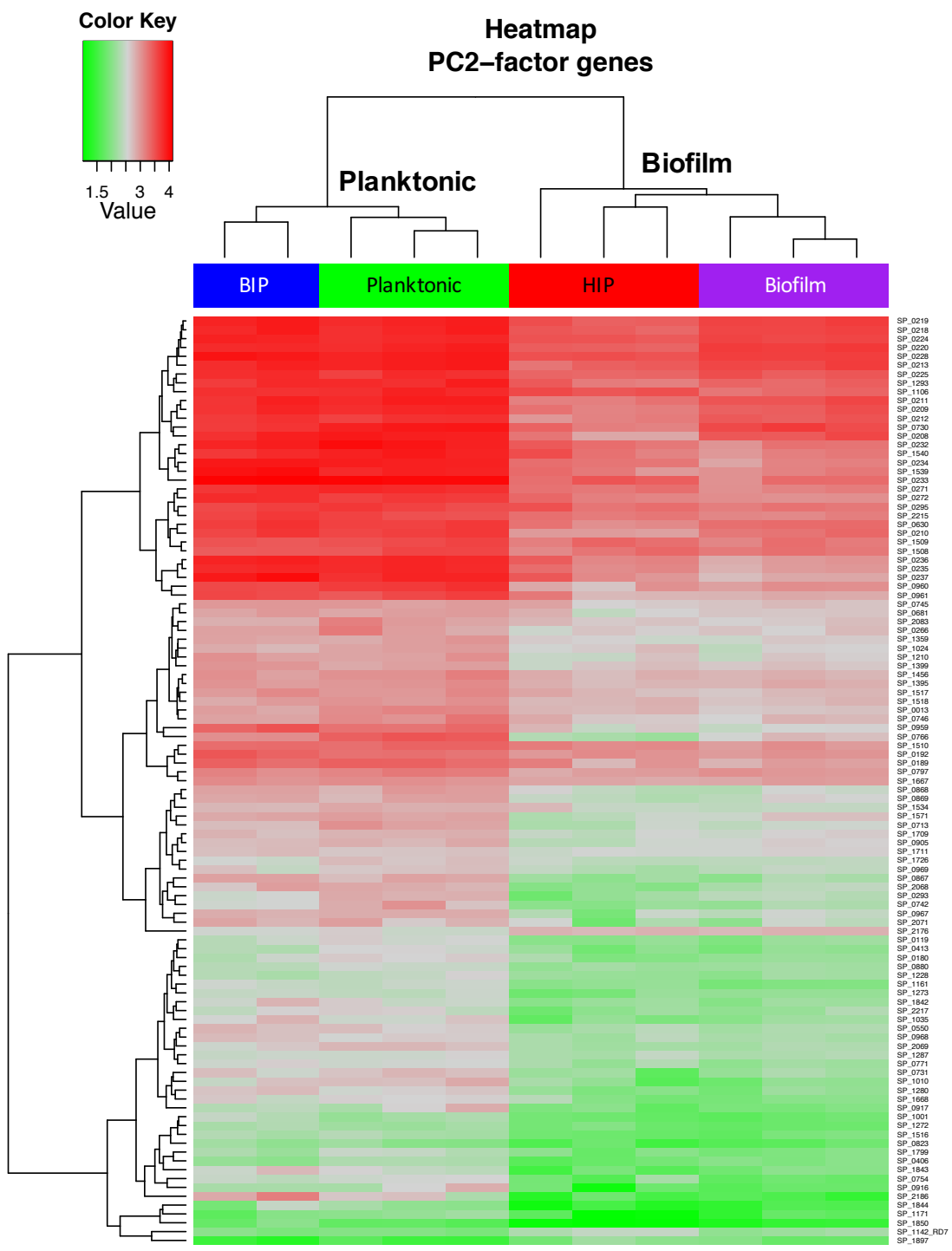

Supplement: S8 Fig — Heat map depiction of log10(RPKM) gene expressions levels for genes that drive the separation of planktonic (in vitro and BIP) and biofilm (in vitro and HIP) populations along the PC2 Y-axis of Fig 5A. 105 genes correlated at ≥85% with PC2. (PDF) [file ppat.1006582.s008.pdf]

### Figure S9

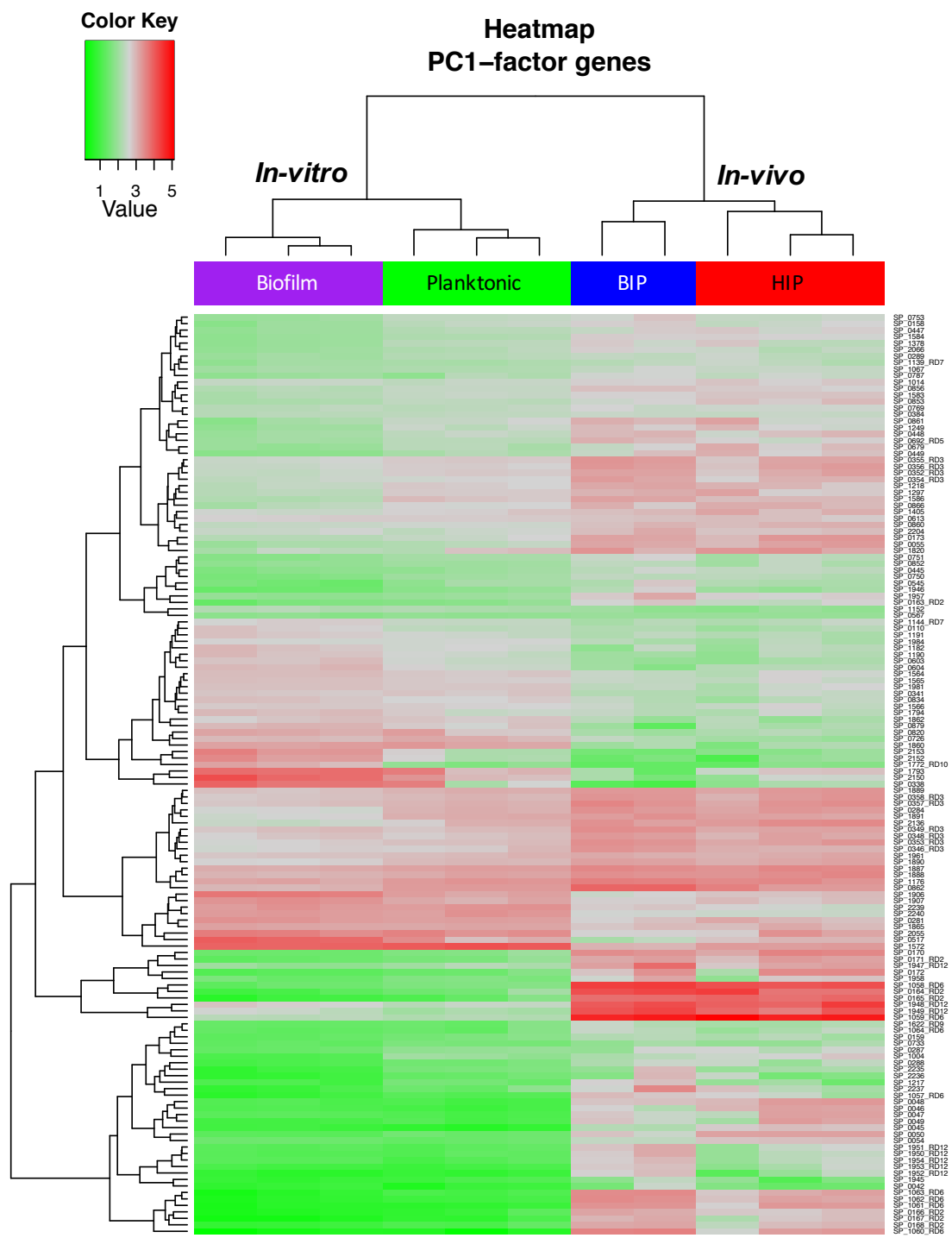

Supplement: S9 Fig — Heat map depiction of log10(RPKM) gene expressions levels for genes that drive the separation of in vitro (biofilm and planktonic) and in vivo (HIP and BIP) populations along the PC1 X-axis of Fig 5A. 142 genes correlated at ≥85% with PC1. Note that genes within RD2, RD6 and RD12 were among the major PC1-correlated genes. (PDF) [file ppat.1006582.s009.pdf]

Fig S11

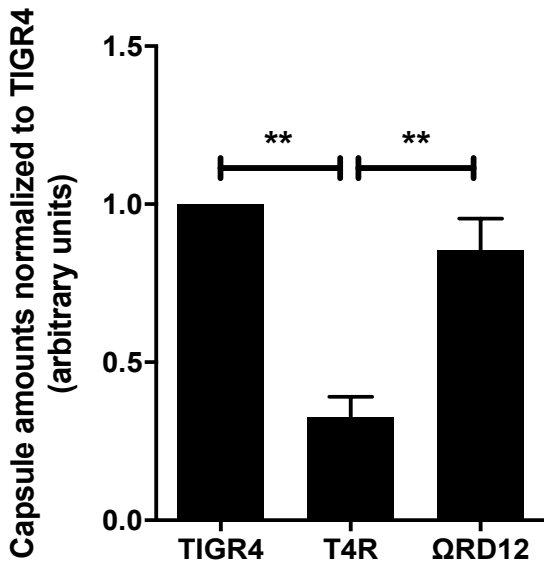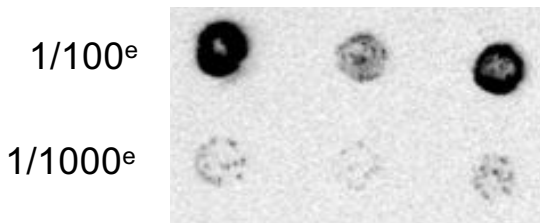

Supplement: S11 Fig — Normalized densitometric quantification of capsule levels by expressed by equal biomass of planktonic wildtype TIGR4 (n = 3), planktonic T4ΩRD12 (ΩRD12) (n = 3) is provided. An isogenic capsule deficient TIGR4 strain (T4R) was tested as the negative control. Statistical analysis was performed by comparison of capsule levels from planktonic T4ΩRD12 and planktonic T4R to planktonic- wildtype TIGR4 (n = 3) using One-way ANOVA. Representative Immunodot blot for capsule levels is shown. (PDF) [file ppat.1006582.s011.pdf]

**Fig S12**

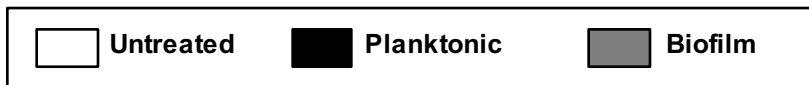

**A**

**J774A.1 Macrophages**

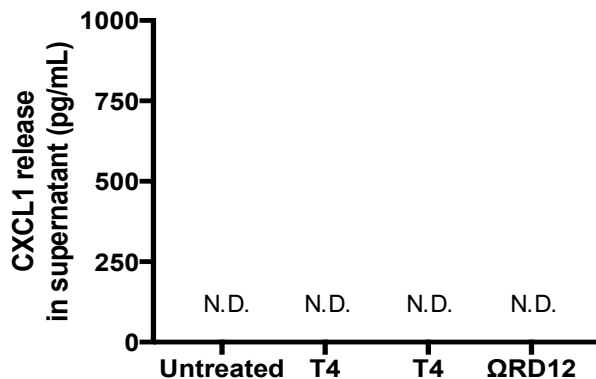

**HL-1 Cardiomyocytes**

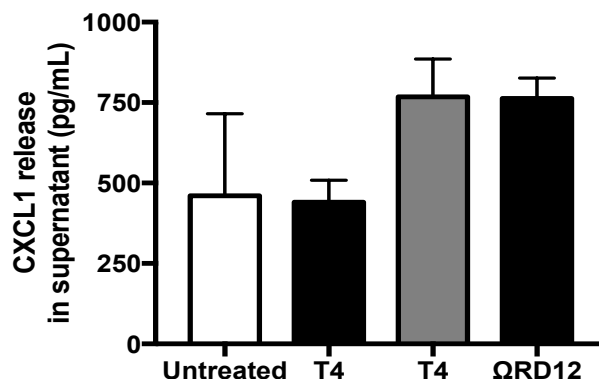

**B**

**J774A.1 Macrophages**

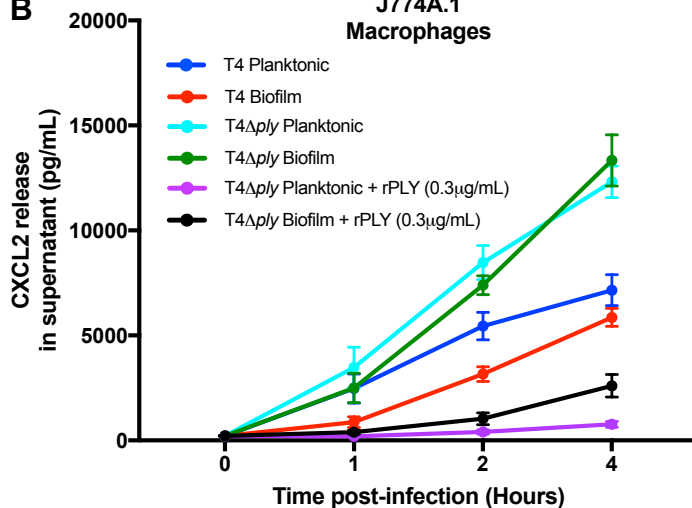

**C**

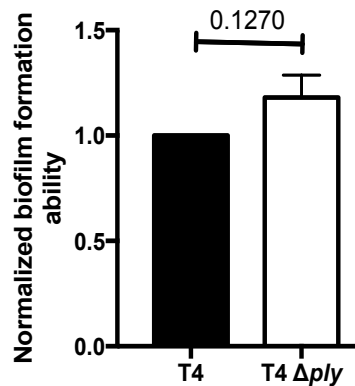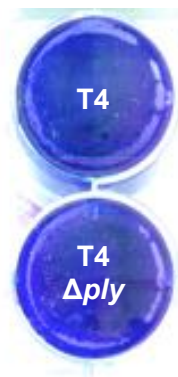

Supplement: S12 Fig — (A) Macrophages and cardiomyocytes do not produce CXCL1 in response to pneumococcal challenge. CXCL1 cytokine production by J774A.1 macrophages and HL-1 cardiomyocytes following 4 hour exposure to equal biomass of planktonic- and biofilm- TIGR4 (T4) or the RD12 deficient mutant strain. N.D. denotes not detectable. Statistical analysis was performed using non-parametric One-way ANOVA (Kruskal-Wallis Test); no significant CXCL1 production was observed compared to untreated cells. (B) Biofilm pneumococci mute the macrophage CXCL2 response in a pneumolysin-dependent manner. CXCL2 production by J774A.1 macrophages at designated time points following exposure to equal biomass of planktonic-, biofilm- TIGR4 (T4), planktonic-, biofilm- T4 Δply and planktonic-, biofilm- T4 Δply complemented with exogenous recombinant pneumolysin (rPLY) at a concentration 0.3μg/mL. Experiments were performed as three biological replicates with 3 technical replicates each. Statistical analysis was performed using ordinary one-way ANOVA. (C) Pneumolysin is not required for biofilm formation. Static biofilm-forming ability of TIGR4 (T4) and it isogenic pneumolysin deficient mutant (T4 Δply) was assessed in a 48-hour 6-well polystyrene plate model (n = 5 experiments). Biofilm biomass was measured using crystal violet staining. Statistical analysis was performed using Student’s t-test. No statistically significant difference was observed. Representative crystal violet stained biofilms are shown. Data are represented as mean ± SEM. (PDF) [file ppat.1006582.s012.pdf]

**Fig S13**

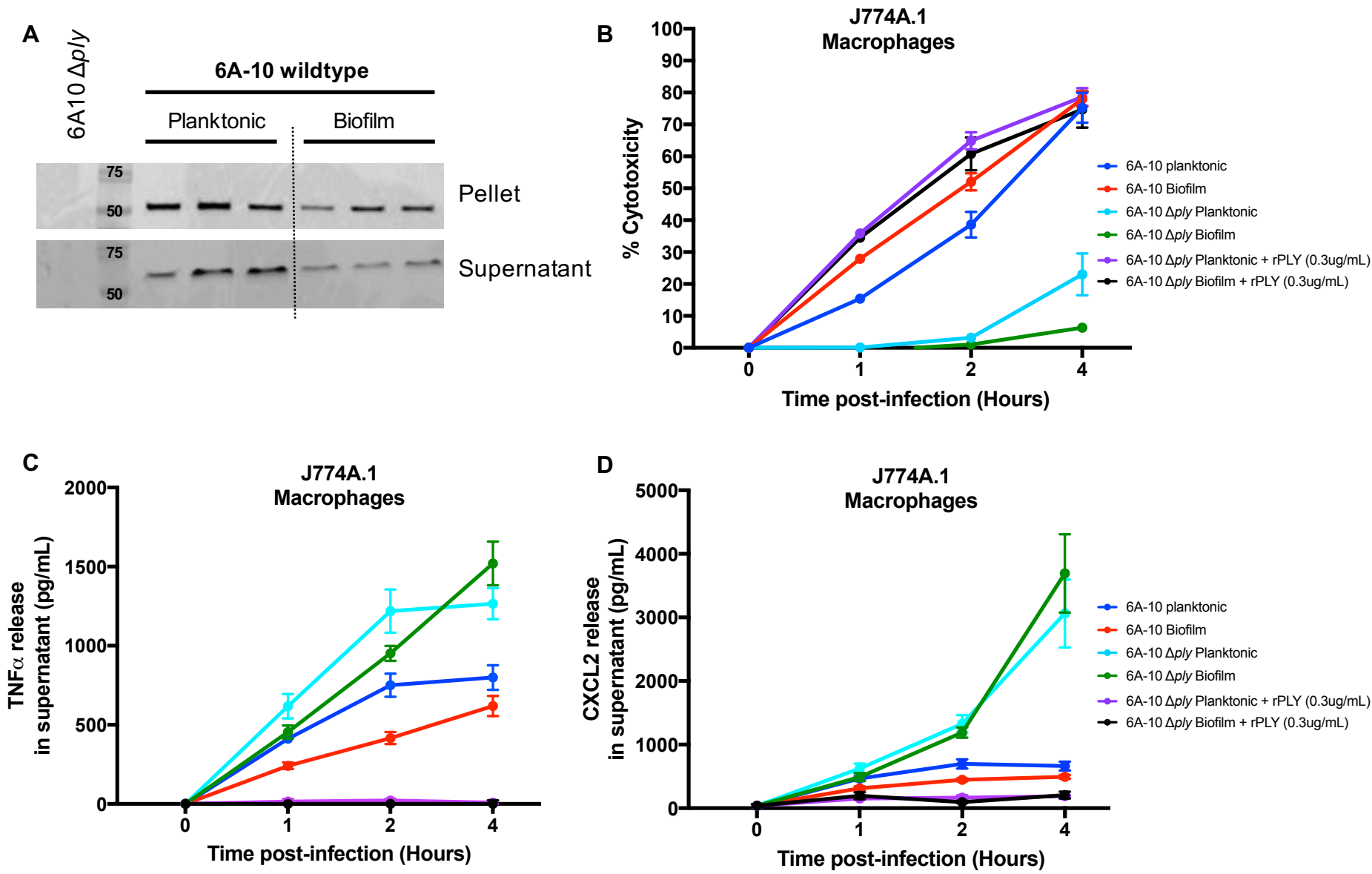

Supplement: S13 Fig — (A) Western blots for pneumolysin levels in equal biomass of whole cell lysates (pellets) and supernatants of planktonic- wildtype 6A-10 (n = 3), and biofilm- wildtype 6A-10 (n = 3). An isogenic pneumolysin deficient 6A-10 strain (6A-10 Δply) was tested as the negative control. (B) LDH release cytotoxicity assay of J774A.1 macrophages challenged with equal biomass of planktonic-, biofilm- 6A-10, planktonic-, biofilm- 6A-10 Δply and planktonic-, biofilm- 6A-10 Δply complemented with exogenous recombinant pneumolysin (rPLY, 0.3μg/mL) as determined at 0, 1, 2, 4 hours post-infection (n = 3 biological replicates, each with 3 technical replicates). Statistical analysis was performed using ordinary one-way ANOVA. (C) TNFα production by J774A.1 macrophages at designated time points following exposure to an equal biomass of planktonic-, biofilm- 6A-10, planktonic-, biofilm- 6A-10 Δply and planktonic-, biofilm- 6A-10 Δply complemented with exogenous recombinant pneumolysin (rPLY, 0.3μg/mL) as determined at 0, 1, 2, 4 hours post-infection (n = 3 biological replicates, each with 3 technical replicates). Statistical analysis was performed using ordinary one-way ANOVA. (D) CXCL2 production by J774A.1 macrophages at designated time points following exposure to an equal biomass of planktonic-, biofilm- 6A-10, planktonic-, biofilm- 6A-10 Δply and planktonic-, biofilm- 6A-10 Δply complemented with exogenous recombinant pneumolysin (rPLY, 0.3μg/mL) as determined at 0, 1, 2, 4 hours post-infection (n = 3 biological replicates, each with 3 technical replicates). Statistical analysis was performed using ordinary one-way ANOVA. (PDF) [file ppat.1006582.s013.pdf]

Fig S14

A

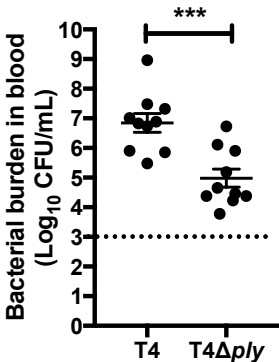

B

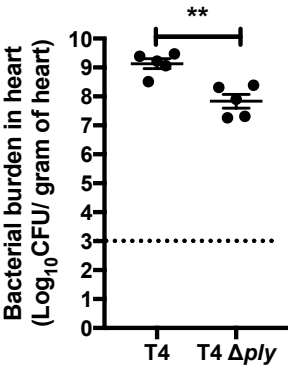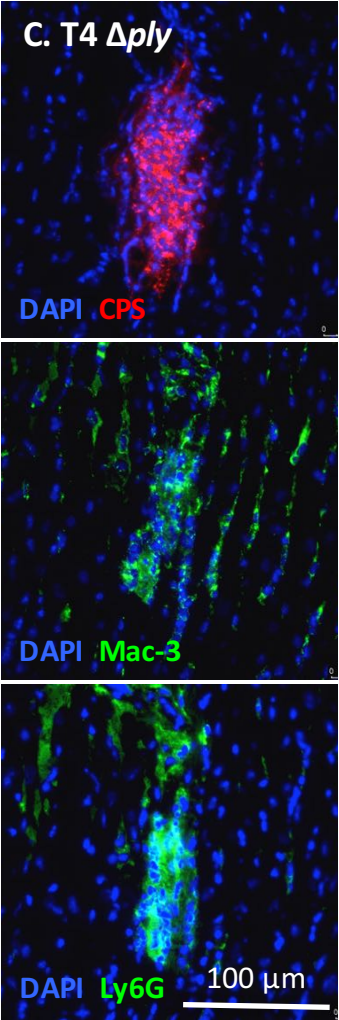

D

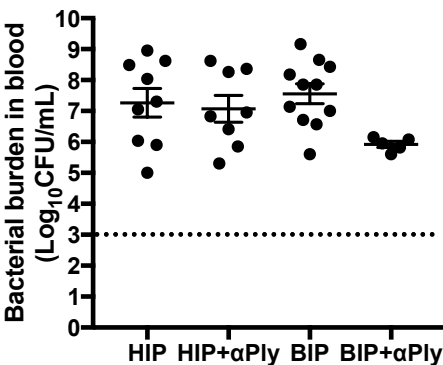

E

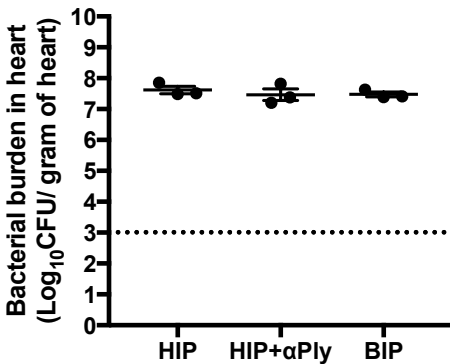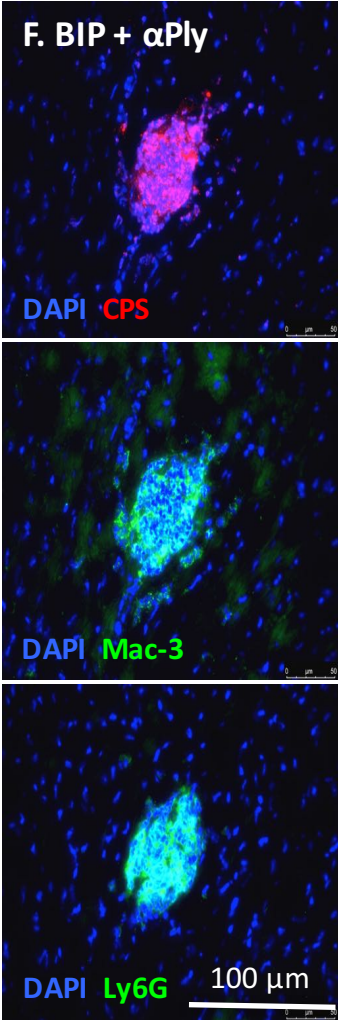

Supplement: S14 Fig — (A, B) Pneumococcal titers in the (A) blood and (B) heart of mice infected with TIGR4 (T4) and T4 Δply 30 hours post-infection. Mann-Whitney test comparing the mutant strain titers to the wildtype TIGR4 titers was performed. (C) Representative high magnification immunofluorescent microscopy images of cardiac microlesions from mice infected with T4 Δply 30 hours post infection, showing presence of: capsule (stained with anti-serotype 4 capsule antibody [CPS], red), cardiac macrophages (stained using anti-Mac-3 antibody [Mac-3], green), and infiltrated neutrophils (stained with anti-Ly-6G antibody [Ly-6G], green). A minimum of 4 stained heart sections were examined. (D, E) Pneumococcal titers in the (D) blood and (E) hearts of naive and passively immunized (αPly)- mice infected with HIP or BIP 30 hours post infection. Mann-Whitney test comparing the bacterial burdens to the wildtype TIGR4 titers was performed. No statistically significant difference was observed. P value: ** ≤ 0.01, *** ≤ 0.001 Data are represented as mean ± SEM. (F) Representative high magnification immunofluorescent microscopy images of cardiac microlesions from passively immunized (αPly)- infected with BIP 30 hours post infection, showing presence of: capsule (stained with anti-serotype 4 capsule antibody [CPS], red), cardiac macrophages (stained using anti-Mac-3 antibody [Mac-3], green), and infiltrated neutrophils (stained with anti-Ly-6G antibody [Ly-6G], green). A minimum of 4 stained heart sections were examined. (PDF) [file ppat.1006582.s014.pdf]
