# Supplementary material for: Streptococcus pneumoniae in the heart subvert the host response through biofilm-mediated resident macrophage killing
Source: PLoS Pathog. 2017 Aug 25;13(8):e1006582. doi: 10.1371/journal.ppat.1006582 (PMC5589263; doi:10.1371/journal.ppat.1006582)
Supplement: S5 Fig — Representative tile-stitched image of whole heart sections from uninfected control mice (n = 3), TIGR4 infected mice (n = 3), myocardial infarcted mice (n = 1) and sham surgery mice (n = 1). The cardiac sections (stained with DAPI, blue) were probed for TIGR4 (red), using serotype 4 capsule polysaccharide antisera, and for exposed galactose residues (green), using fluorescein labeled Erythrina crystagalli lectin, within the heart. Sterile tissue injury due to experimentally induced myocardial infarction did not result in galactose exposure as observed during TIGR4- mediated microlesion formation. (PDF) [file ppat.1006582.s005.pdf]

Fig S5

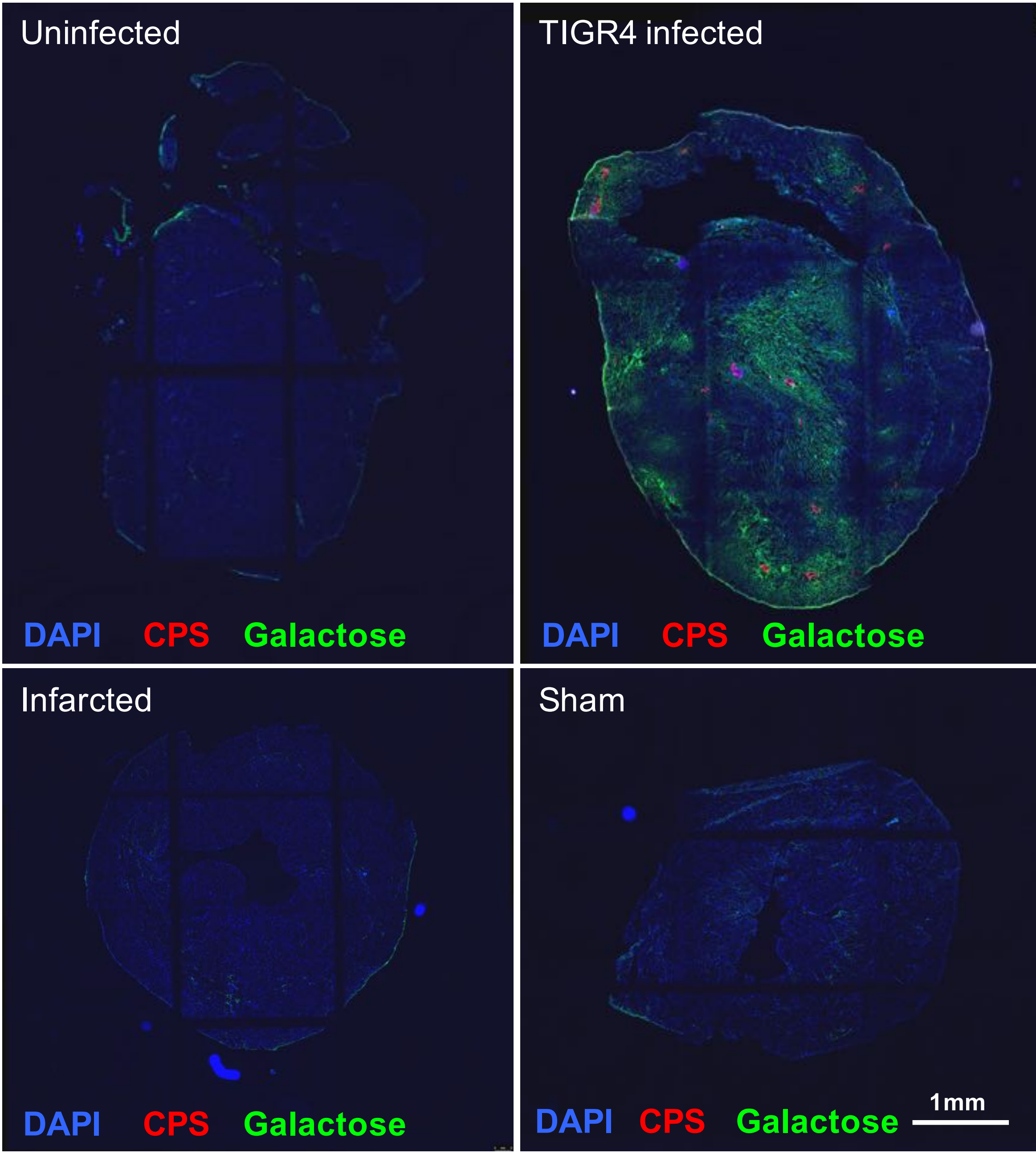
Uninfected
DAPI
CPS
Galactose
TIGR4 infected
DAPI
CPS
Galactose
Infarcted
DAPI
CPS
Galactose
Sham
1mm
DAPI
CPS
Galactose
