# Supplementary material for: Streptococcus pneumoniae in the heart subvert the host response through biofilm-mediated resident macrophage killing
Source: PLoS Pathog. 2017 Aug 25;13(8):e1006582. doi: 10.1371/journal.ppat.1006582 (PMC5589263; doi:10.1371/journal.ppat.1006582)
Supplement: S7 Fig — Gene expression fold change levels for (A) in vitro biofilms, (B) Heart- isolated pneumococci (HIP), and (C) blood Isolated pneumococci (BIP) each in comparison with planktonic pneumococci as determined using RNA-Seq analysis were confirmed using qRT-PCR for 69 candidate genes. Expression fold change values obtained by RNA-seq analysis (X-axis) and qRT-PCR (Y-axis) are in good agreement as evidenced from strong correlation coefficients. The primer sequences used for qRT-PCR are provided is S3 Table. (PDF) [file ppat.1006582.s007.pdf]

Fig S7

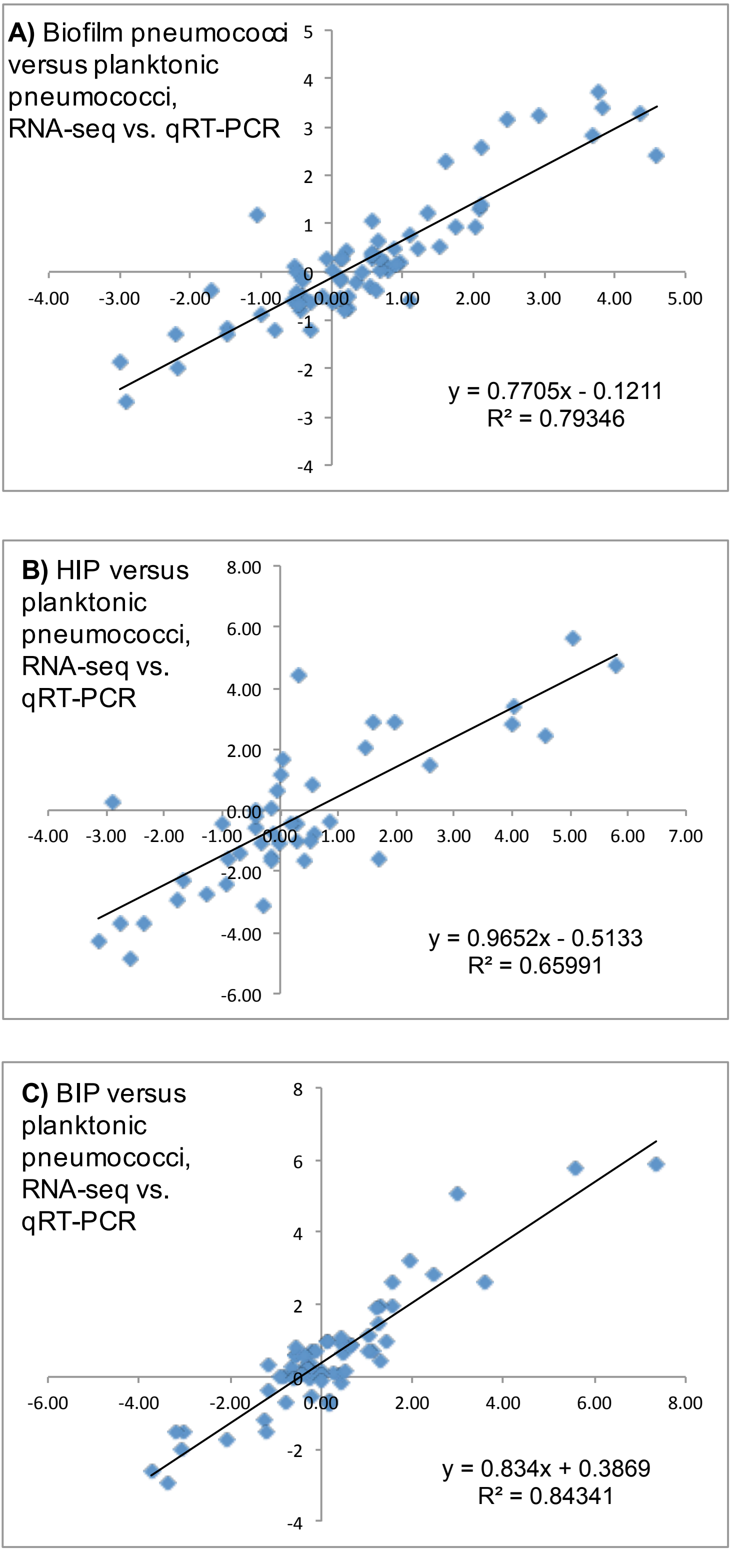

A) Biofilm pneumococci versus planktonic pneumococci, RNA-seq vs. qRT-PCR
5
4
3
2
1
0
-1
-2
-3
-4
-4.00
-3.00
-2.00
-1.00
0.00
1.00
2.00
3.00
4.00
5.00
y = 0.7705x - 0.1211
R² = 0.79346
B) HIP versus planktonic pneumococci, RNA-seq vs. qRT-PCR
8.00
6.00
4.00
2.00
0.00
-2.00
-4.00
-6.00
-4.00
-3.00
-2.00
-1.00
0.00
1.00
2.00
3.00
4.00
5.00
6.00
7.00
y = 0.9652x - 0.5133
R² = 0.65991
C) BIP versus planktonic pneumococci, RNA-seq vs. qRT-PCR
8
6
4
2
0
-2
-4
-6.00
-4.00
-2.00
0.00
2.00
4.00
6.00
8.00
y = 0.834x + 0.3869
R² = 0.84341
