# Supplementary material for: Streptococcus pneumoniae in the heart subvert the host response through biofilm-mediated resident macrophage killing
Source: PLoS Pathog. 2017 Aug 25;13(8):e1006582. doi: 10.1371/journal.ppat.1006582 (PMC5589263; doi:10.1371/journal.ppat.1006582)

Fig S10

A

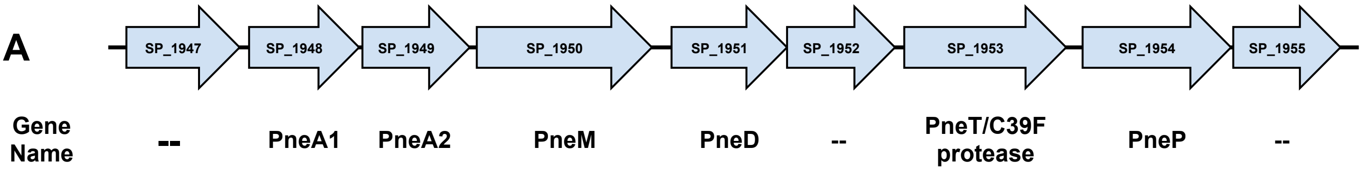

Gene Name	Predicted function
Pne A1/A2	Pneumococcin A
PneM	Pneumococcin A modifying protein
PneD	FMN Reductase<br>(Putative Pneumococcin A oxidative decarboxylase)
PneT/C39F protease	Pneumococcin A transporter
PneP	Pneumococcin A specific protease
--	Unknown function

B

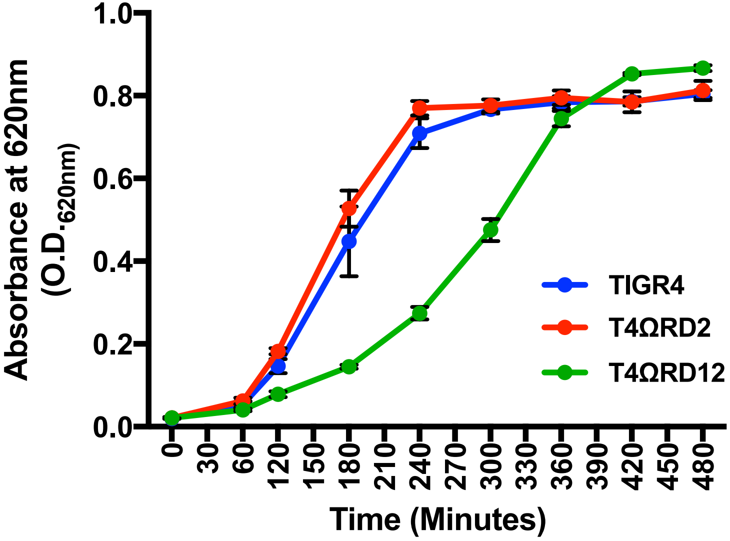

C

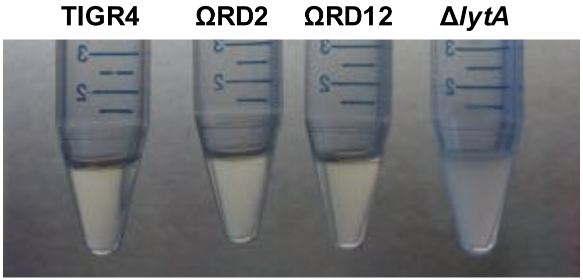

Supplement: S10 Fig — (A) Organization of genes within RD12. Putative gene names and predicted functions are shown. (B) T4ΩRD12 exhibits no long-term growth defects. Growth curves of TIGR4, T4ΩRD2, and T4ΩRD12 in Todd Hewitt Broth with 0.5% yeast extract (THY) are presented. Despite a delayed log phase growth T4ΩRD12 showed no long-term growth defects. Experiments were performed in triplicates. (C) T4ΩRD12 undergoes normal autolysis during bile solubility assay. Comparative autolytic properties of TIGR4, ΩRD2, ΩRD12 and ΔlytA (negative control) isogenic mutants on treatment with pneumococcal lysis buffer (0.01% SDS, 0.1% DOC, AND 0.015 M Na-citrate) are shown. The lytA deletion mutant was a kind gift from Dr. Terry Brissac, personal communication. (PDF) [file ppat.1006582.s010.pdf]
